# *Isodon lophanthoides* alleviates liver fibrosis via modulation of purine metabolism and NF-κB signaling pathway: insights from multi-omics analysis

**DOI:** 10.3389/fphar.2025.1630927

**Published:** 2025-08-01

**Authors:** Kuikui Chen, Jie Liang, Yong Tan, Yaohua Li, Xiaojiao Pan, Zhonghui Guo, Wentao Zhang, Zongxi Sun

**Affiliations:** ^1^ School of Pharmacy, Guangxi University of Chinese Medicine, Nanning, Guangxi, China; ^2^ Guangxi Zhuang Yao Key Laboratory of Medicine, Guangxi University of Chinese Medicine, Nanning, Guangxi, China; ^3^ Department of Food Science and Human Nutrition, Citrus Research and Education Center, University of Florida, Lake Alfred, FL, United States; ^4^ Teaching Experiment Training Center, Guangxi University of Chinese Medicine, Nanning, Guangxi, China; ^5^ Ruikang Clinical Medical School, Guangxi University of Chinese Medicine, Nanning, Guangxi, China

**Keywords:** Isodon lophanthoides, liver fibrosis, multi-omics, qualitative and quantitative analysis, metabolomics

## Abstract

**Background:**

*Isodon lophanthoides*, a core botanical drug in Yao ethnomedicine, has traditionally been used to treat jaundice-type hepatitis and cholecystitis. However, its therapeutic potential and mechanisms against liver fibrosis remain largely unexplored.

**Methods:**

The metabolites of *I. lophanthoides* water extract (ILW) were characterized by ultra-performance liquid chromatography (UPLC) and UPLC coupled with quadrupole time-of-flight mass spectrometry (Q-TOF/MS). A carbon tetrachloride (CCl_4_)-induced liver fibrosis mouse model was employed to evaluate the anti-fibrotic effects of ILW. An integrated multi-omics approach encompassing transcriptomics, proteomics, and metabolomics was used to elucidate the underlying mechanisms, further supported by Western blotting, targeted metabolite quantification, and enzyme-linked immunosorbent assay (ELISA).

**Results:**

Thirty-two metabolites were identified in ILW. Among them, the concentrations of caffeic acid, rosmarinic acid, schaftoside, and isoschaftoside were determined to be 1.10, 5.13, 0.12, and 0.18 mg·g^-1^, respectively. ILW treatment significantly reduced serum levels of alanine aminotransferase (ALT), aspartate aminotransferase (AST), procollagen type III (PC-III), collagen type IV (COL-IV), laminin (LN), and hyaluronic acid (HA) in liver fibrotic mice. Histopathological analyses showed that ILW significantly alleviated liver inflammation, collagen deposition, and fibrosis. Multi-omics analysis revealed that ILW’s anti-fibrotic effects are linked to modulation of purine metabolism and inhibition of the nuclear factor kappa-B (NF-κB) signaling pathway. High-dose ILW lowered hepatic levels of adenine, adenosine monophosphate (AMP), and inosine monophosphate (IMP), while increasing adenosine, hypoxanthine, and N^6^-methyladenosine (m6A). Furthermore, high-dose and medium-dose ILW downregulated key NF-κB-related proteins, including toll-like receptor 4 (TLR4), myeloid differentiation primary response 88 (MyD88), phosphorylated NF-κB, transforming growth factor beta 1 (TGF-β1), tumor necrosis factor-alpha (TNF-α), interleukin-6 (IL-6), and interleukin-1 beta (IL-1β).

**Conclusion:**

ILW exerts protective effects against liver fibrosis by attenuating inflammation, fibrosis, and liver damage through modulation metabolism modulation and NF-κB pathway inhibition. These findings provide a scientific basis for the traditional use of *I. lophanthoides* in liver-related disorders.

## 1 Introduction

Liver fibrosis is a frequent pathological outcome associated with a range of chronic hepatic conditions, arising from factors such as viral hepatitis, alcohol abuse, drug toxicity, and schistosomiasis (Hammerich and Tacke, 2023; He et al., 2024; Meng et al., 2025). This condition is characterized by excessive accumulation of extracellular matrix (ECM) components, which, if unresolved, may progress to cirrhosis, portal hypertension, or hepatocellular carcinoma (HCC), ultimately resulting in liver failure (Khan and Saxena, 2021). Epidemiological data suggest that approximately 2.85% of adults suffer from advanced liver fibrosis, and cirrhosis is diagnosed in about 0.87% of the population (Man et al., 2023). These conditions are associated with poor clinical outcomes and are responsible for over one million deaths globally each year (Wang et al., 2023a). Despite the pressing burden of liver fibrosis, there are currently no FDA-approved anti-liver fibrotic therapies. Existing treatment strategies focus primarily on hepatoprotection, anti-inflammation, antioxidation, and choleretic effects to manage symptoms and delay disease progression (Chinese Society of Hepatology Chinese Medical Association et al., 2020; Setyawati et al., 2024; Zhou Q. M. et al., 2024). Given the complexity of liver fibrosis pathogenesis, the development of safe and efficacious treatments remains a pressing priority.

*Isodon lophanthoides* (Buch.-Ham. ex D. Don) H. Hara, a species in the Lamiaceae family, is a regionally used botanical drug endemic to southern China, such as Guangdong, Guangxi, and Fujian. It has long been utilized in the traditional medical systems of the Yao ethnic minority to treat hepatobiliary disorders such as jaundice-type hepatitis and cholecystitis. Within Yao medical theory, liver diseases are primarily attributed to the invasion of pathogenic toxins (du xie), which obstruct internal circulation, leading to the accumulation of phlegm and blood stasis, and ultimately resulting in disease manifestation (Wang and Ma, 2024). Known locally as “Xianwen Xiang Cha Cai” or “Xi Huang Cao,” this botanical is classified in Yao ethnomedicine as a “wind-trauma” remedy, employed to clear heat and toxins, dispel dampness, and promote blood circulation. It is mainly used clinically to treat jaundice-type hepatitis and cholecystitis (Sun et al., 2020; Guangxi Food and Drug Administration, 2012). While widely used as both botanical drug and functional food (often in decoctions or soups), it also serves as a key component in Chinese patent medicines such as Xiaoyanlidan tablets. Phytochemical studies have identified various bioactive metabolites in *I. lophanthoides*, including flavonoids, organic acids and diterpenoids, which exhibit immunomodulatory, antioxidant, and antitumor properties (Wong et al., 2016; Khoa et al., 2024; Zhou et al., 2014). However, despite its extensive ethnopharmacological applications, it is not yet included in any national or international pharmacopoeia. Most existing research has focused on the isolation of specific metabolites, quality assessment, or evaluation of pharmacodynamic effects in liver protection and anticancer models (Qin et al., 2024; Qiu et al., 2022; Zhou H. L. et al., 2024; Lin et al., 2019). Preliminary findings from high-throughput and *in vivo* screening suggest that aqueous or flavonoid-rich extracts from this botanical may possess anti-liver fibrotic potential (Huang et al., 2018; Zheng et al., 2017; Zhong et al., 2021). However, the precise mechanisms underlying these effects remain largely unexplored.

As the liver serves as the central hub of metabolic activity, disturbances in metabolic homeostasis, such as dysregulation of purine, lipid, amino acid, and bile acid metabolism, can contribute to hepatic injury and the progression of fibrosis (Xiao et al., 2024; Xiong et al., 2022; Hamanaka and Mutlu, 2021; Zhang et al., 2022). Concurrently, chronic inflammation plays a pivotal role in initiating and sustaining fibrogenesis. Canonical inflammatory pathways, including the NF-κB signaling axis, modulate the expression of pro-inflammatory cytokines that activate hepatic stellate cells (HSCs), while the TGF-β/Smad pathway orchestrates fibrogenic responses by promoting collagen synthesis and inhibiting matrix degradation (Li et al., 2025a; Chen et al., 2023). The crosstalk between metabolic dysfunction and persistent inflammation drives fibrotic progression.

**FIGURE 1:**
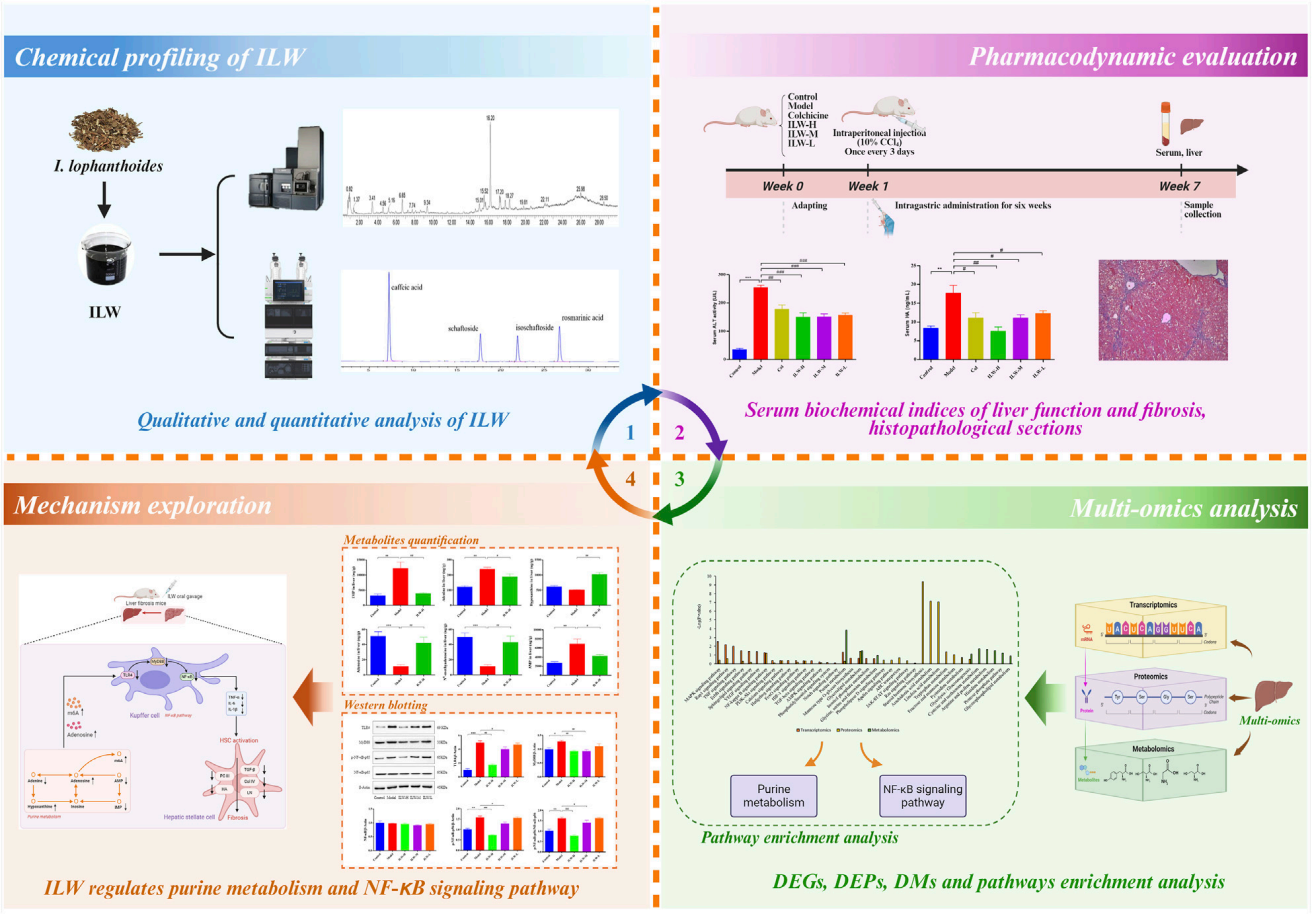
The workflow of the integrated analysis strategy in this study.

To address the current knowledge gap, we hypothesized that the water extract of *I. lophanthoides* (ILW) exerts anti-liver fibrotic effects through the modulation of specific metabolic and inflammatory pathways. Multi-omics approaches offer a powerful platform for comprehensively deciphering the pharmacodynamic actions and molecular targets of botanical drugs, as they allow for the dynamic monitoring of gene, protein, and metabolite alterations during therapeutic intervention (Dong et al., 2022; Ma et al., 2024; Tie et al., 2024; Zhang et al., 2024). This study aimed to systematically evaluate the anti-liver fibrotic activity of ILW using a carbon tetrachloride (CCl_4_)-induced liver fibrosis model in mice, and to elucidate its mechanisms of action through an integrated multi-omics approach. As illustrated in Figure 1, the chemical profile of ILW was characterized and quantified using UPLC-Q-TOF/MS and UPLC techniques. Subsequently, the therapeutic efficacy of ILW was assessed in CCl_4_-induced fibrotic mice. Liver tissue samples were subjected to transcriptomic, proteomic, and metabolomic analyses to identify differentially expressed genes, proteins, and metabolites, as well as key signaling pathways affected by ILW treatment. Further, Quantitative profiling of key metabolites and pathway-associated proteins offered deeper mechanistic insights derived from the multi-omics profiling. These findings will contribute valuable foundational evidence supporting the potential clinical utility and product development of *I. lophanthoides* in liver fibrosis management.

## 2 Materials and methods

### 2.1 Reagents

Colchicine tablets (Lot number: 220204) were purchased from Banna Pharmaceutical Co., Ltd. Alanine aminotransferase (ALT) kit (Lot number: 20231024), aspartate aminotransferase (AST) kit (Lot number: 20231024), type IV collagen (COL-Ⅳ) kit (Lot number: 20231123), type III procollagen (PC-III) kit (Lot number: 20231123), laminin (LN) kit (Lot number: 20231123), and hyaluronic acid (HA) kit (Lot number: 20231123) were provided by Nanjing Jiancheng Bioengineering Institute (Nanjing, China). Vitexin (Lot number: RFS-M02302002022, purity >98%), isovitexin (Lot number: RFS-Y11602107028, purity >98%), protocatechuic acid (Lot number: RFS-Y03111812016, purity >98%), rosmarinic acid (Lot number: AF22022152, purity >98%), schaftoside (Lot number: AF21061501, purity >98%), isoschaftoside (Lot number: AF21062602, purity >98%), and vicenin-2 (Lot number: AF20112508, purity >98%) were obtained from Chengdu Aifa Biotechnology Co., Ltd. (Chengdu, China). Electrophoresis buffer (Lot number: 20,220,810), electrotransfer buffer (Lot number: 240005008), TBST solution (Lot number: 20230214), Tumor necrosis factor-α (TNF-α) enzyme-linked immunosorbent assay (Elisa) kit (Lot number: CSB-E04741m), interleukin-1 beta (IL-1β) Elisa kit (Lot number: CSB-E8054m), interleukin 6 (IL-6) Elisa kit (Lot number: CSB-E04639m), and transforming growth factor beta 1 (TGF-β1) Elisa kit (Lot number: CSB-E04726m) were provided by Cusabio (Wuhan, China). Primary antibodies against toll-like receptor 4 (TLR4) (Lot number: 19811-1-AP), myeloid differentiation primary response protein 88 (MyD88) (lot number: 23230-1-AP), nuclear factor kappa-B (NF-κB) p65 (Lot number: 10745-1-AP), and Phospho-NF-κB p65 (Lot number: 82335-1-RR) were obtained from Proteintech (Chicago, United States). BCA protein assay kit (Lot number: 040224240722), Primary antibody diluent (Lot number: 011422220520), secondary antibody diluent (Lot number: 112522221205) and ECL chemiluminescence kit (Lot number: 091523231011) were supplied by Biyotime Biotechnology (Shanghai, China).

### 2.2 Botanical drug

The plant material was sourced from Guangxi Xianzhu Traditional Chinese Medicine Technology Co., Ltd. (Nanning, China). It was authenticated by Professor Yong Tan of Guangxi University of Chinese Medicine as the dried aerial portions of *Isodon lophanthoides* (Buch.-Ham. ex D. Don) H. Hara, belonging to the Lamiaceae family (Supplementary Table S1). A reference specimen (voucher no. GUCM2021013) has been archived in the Department of Drug Analysis at Guangxi University of Chinese Medicine. The collection and use of plant materials complied fully with the Nagoya Protocol on Access and Benefit Sharing, the Convention on International Trade in Endangered Species of Wild Fauna and Flora (CITES), and all relevant phytosanitary and biodiversity regulations. The plant species used (*Isodon lophanthoides* (Buch.-Ham. ex D. Don) H. Hara) is not listed under CITES and was obtained from a legal commercial source in China. No international transport of plant materials was involved.

### 2.3 Preparation for ILW

The plant material used in this study was *Isodon lophanthoides* (Buch.-Ham. ex D. Don) H. Hara [Lamiaceae; Herba Isodonis Lophanthoidis], traditionally used in Yao medicine. A total of 10 kg of dried aerial parts were extracted with 80 L of purified water. The extraction process involved two consecutive decoctions, each lasting 1.5 h. The supernatant from each decoction was filtered through 200-mesh gauze, combined, and concentrated under reduced pressure, yielding 1.2 kg of ILW. This product (Batch No. 202209013; Production date: 12 September 2022; Best before: 11 September 2025) is a research-grade aqueous extract developed for pharmacological investigation. It is not marketed as a finished pharmaceutical product and is not subject to national drug regulatory approval. Extraction and sample preparation were conducted by the Preparation R&D Center, Guangxi Hospital of Traditional Chinese Medicine (Nanning, China). No traditional processing methods (e.g., steaming, frying, roasting) were applied prior to extraction.

### 2.4 Qualitative analysis of ILW

#### 2.4.1 Preparation of ILW analytical sample

An accurately measured 100 mg portion of ILW extract was dissolved in 10 mL of methanol and subjected to ultrasonic treatment at 150 W and 50 kHz for 30 min. Then, the final test solution was obtained after filtering via a 0.22 μm membrane.

#### 2.4.2 UPLC-Q-TOF/MS condition

Qualitative analysis was conducted by an UPLC I-Class system coupled with a G2 QTof mass spectrometer. Separation was performed using ACQUITY PRM HSS T3 FIT column (100 mm × 2.1 mm, 1.8 μm). The mobile phases consisted of 0.1% formic acid in water (A) and acetonitrile (B). The gradient elution was set: 0–3 min, 5% B; 3–10 min, 5%−10% B; 10–20 min, 10%–30% B; 20–30 min, 30%–65% B. A 2 μL injection volume was used, with a flow rate of 0.3 mL min^-1^, and the column held at 40°C. In addition, a PDA detector was used for detection, and three detection wavelengths including 254 nm, 270 nm, 300 nm were set.

Mass detection was conducted in positive and negative ionization modes. Spectral data were collected across an *m/z* range of 50–1,500. Capillary voltages were set at 3.0 kV (+) and 2.5 kV (−). The cone voltage was adjusted to 40 V. Cone and desolvation gas were supplied at 50 L h^-1^ and 600 L h^-1^, respectively. Argon was used as the collision gas at 0.15 mL min^-1^. The ion source temperature was kept at 120°C, while the desolvation temperature was 300°C. Collision energies were programmed at 6 eV for low-energy scans and ranged from 20 to 35 eV for the high-energy mode.

#### 2.4.3 Data post-processing strategy

A comprehensive chemical database for *I. lophanthoides* was constructed based on literature reports. Candidate metabolites were rapidly screened using the UNIFI platform. Further identification and confirmation were carried out through an integrated approach, combining reference standards, elemental analysis, fragment ion interpretation, mass spectral comparison, and validation against existing literature.

### 2.5 Quantitative and fingerprint analysis of ILW

Quantification of key metabolites in ILW, including caffeic acid, rosmarinic acid, schaftoside, and isoschaftoside, was carried out by an Agilent 1290 UPLC system. Calibration curves were established for each analyte to ensure accurate measurement. Specific protocols for sample processing, instrumental settings and selection of metabolites for quantification are detailed in the Supplementary Material. To assess batch-to-batch consistency, chromatographic fingerprinting was performed on 12 independent ILW batches using UPLC method. Data analysis was carried out with the Similarity Evaluation System for Chromatographic Fingerprint of Traditional Chinese Medicine (Version 2012.130723, National Pharmacopoeia Commission of China), as detailed in the Supplementary Material.

### 2.6 Pharmacodynamics studies

#### 2.6.1 Preparation of colchicine solution and CCl4 olive oil solution

A colchicine tablet was finely pulverized using a clean, dry mortar. The resulting powder was then suspended in 50 mL of a 0.1% aqueous sodium carboxymethyl cellulose (CMC) solution to formulate the colchicine solution for the positive control group. For the CCl_4_-olive oil solution, 4 mL of CCl_4_ was accurately measured using a pipette, and 36 mL of olive oil was added. The mixture was thoroughly stirred to prepare a 10% CCl_4_-olive oil solution, ready for use.

#### 2.6.2 Animal experiments

SPF-grade KM mice (20 ± 2 g, equal numbers of males and females) were provided by Changsha Tianqin Biotechnology Co., Ltd. Prior to commencement of the experiment, animals were housed under controlled conditions and acclimatized for 7 days. All animal-related procedures received prior approval from the Animal Ethics Committee of the Experimental Center at Guangxi University of Chinese Medicine (No. 2021AC19102) and were conducted in compliance with institutional ethical standards. Following body weight measurement, the mice were randomly allocated into six groups (n = 12): a control group, a model group, a colchicine-treated positive control group (Col), and three ILW treatment groups receiving high (ILW-H), medium (ILW-M), and low (ILW-L) doses, respectively. All mice, except those in the control group, were administered an intraperitoneal injection of 10% carbon tetrachloride (CCl_4_) diluted in olive oil at a dose of 4 mL kg^-1^, once every 3 days for a total duration of 6 weeks to induce liver fibrosis. From the initiation of CCl_4_ administration, mice in the control and model groups were given 0.1% sodium carboxymethyl cellulose (CMC) solution orally (20 mL kg^-1^/day), while the ILW treatment groups received daily intragastric doses of ILW at 1.88 g kg^-1^ (high), 0.94 g kg^-1^ (medium), or 0.47 g kg^-1^ (low). The Col group was given colchicine at 0.20 mg kg^-1^/day. All treatments lasted for six consecutive weeks. Upon completion of the final dose, the animals were denied food for 12 h but given free access to water. Blood was collected from the orbital sinus into centrifuge tubes and spun at 2000 rpm for 10 min. Serum was carefully separated and stored at −80°C until analysis. After mice were euthanized via cervical dislocation, liver tissue was promptly harvested, rinsed in saline and gently blotted. The left hepatic lobe was fixed in 4% paraformaldehyde, while the remaining tissue was stored at −80°C for subsequent assays.

#### 2.6.3 Assessment of serum biochemical markers

ALT and AST levels in serum were determined by microplate-based enzymatic assays. ELISA were employed to quantify the concentrations of HA, LN, PC-III, and COL-IV, strictly adhering to protocols provided by the reagent manufacturers.

#### 2.6.4 Histopathological evaluation

Liver specimens were fixed in 4% paraformaldehyde, processed through standard dehydration, embedded in paraffin, and sectioned into slices. These sections were dewaxed, rehydrated, and stained using hematoxylin and eosin (HE) and Masson’s trichrome techniques. Pathological changes and fibrosis in hepatic tissue were assessed under a light microscope.

### 2.7 Transcriptomics analysis

Liver tissues from control, model, and ILW-H groups (n = 3) were retrieved from −80°C. After total RNA was extracted, the concentration and integrity were assessed by a Qubit 4.0 fluorometer and Qsep400 bioanalyzer, respectively. Poly(A) mRNA was enriched using AMPure XP beads, fragmented, and reverse-transcribed into cDNA. After end-repair, adapter ligation, and 15 PCR cycles, libraries (∼300 bp) were constructed and validated using an Agilent 2100 Bioanalyzer, then sequenced using Illumina platform. Then, raw reads were filtered, aligned to the reference genome, and quantified. DESeq2 (v1.38.3) was used to identify differentially expressed genes (DEGs) with |log_2_Fold Change (FC)| > 0.58 and FDR <0.05. Up- and downregulated genes were defined by FC > 1.5 and <0.67, respectively. Venny 2.1 (https://bioinfogp.cnb.csic.es/tools/venny/) was employed to visualize DEG overlaps, and Kyoto Encyclopedia of Genes and Genomes (KEGG) pathway enrichment was conducted by clusterProfiler in R (v4.3.1). Data visualization was carried out using the Bioinformatics Platform (https://www.bioinformatics.com.cn/). The transcriptomic and proteomic datasets have been deposited in the Zenodo platform. Accession number is 15460165.

### 2.8 Label-free quantitative proteomics analysis

Liver tissues (n = 3 per group from control, model, and ILW-H cohorts) were pulverized in liquid nitrogen. The homogenized powder was lysed in buffer (8 M urea, 1 mM PMSF, 2 mM EDTA), followed by sonication and centrifugation (15,000 rpm, 10 min, 4°C). Then, protein concentrations quantified using a BCA assay. Proteins were digested overnight at 37°C using trypsin in 25 mM ammonium bicarbonate. Peptides were acidified (pH 2–3) with 20% TFA, purified via C18 cartridges, and quantified using the Pierce™ peptide detection kit. Peptide mixtures were resolved on a Vanquish Neo UHPLC system using a dual-column configuration (PepMap Neo trap and analytical columns). Mass spectra were acquired on an Orbitrap Astral MS in positive ion mode. Survey scans covered an *m/z* range of 380–980 at a resolution of 240,000. DIA acquisition used 299 variable isolation windows (2 Th), HCD at 25%, and dynamic accumulation (AGC target 500%, maximum IT 3–5 ms).

Raw spectral data were analyzed using Proteome Discoverer 2.5. Protein identification was performed using DIA-NN (v1.8.1). A 1% FDR cutoff was applied for confident protein assignment. Differentially expressed proteins (DEPs) analysis used a threshold of *p* < 0.05 and FC > 1.2 or <0.83. Proteins exceeding a FC of 1.2 were deemed upregulated, while those below 0.83 were downregulated. Overlapping DEPs across group comparisons (model vs. control, ILW-H vs. model) were visualized using Venny 2.1. KEGG pathway enrichment was carried out vis KOBAS (http://kobas.cbi.pku.edu.cn/), and results were graphically rendered via the Bioinformatics online platform.

### 2.9 Metabolomics analysis

Liver tissues (n = 3 per group from control, model, and ILW-H cohorts) were subjected to metabolomic profiling. Approximately 50 mg tissue was extracted with 500 μL of prechilled 70% methanol (−20°C), and centrifuged at 12,000 rpm for 10 min. A 300 μL aliquot of the supernatant was kept at −20°C for 30 min, then centrifuged again under the same conditions. The final supernatant (200 μL) was passed through a protein precipitation plate prior to analysis using a SCIEX QTRAP 6500+ system. To fully investigate the metabolites, two chromatography modes, T3 column and Amide column, were used for sample separation, and the detail parameters are presented in the Supplementary Material. Mass spectrometry was carried out using ESI in positive and negative modes. The source temperature was 550°C, with ion voltages set at 5.5 kV (+) and 4.5 kV (−). Curtain gas was maintained at 35 psi. Data acquisition used dynamic multiple reaction monitoring (MRM) mode.

Raw data were handled using Analyst 1.6.3 and MultiQuant 3.03 for peak detection, alignment, and integration. Principal component analysis (PCA) and pathway enrichment were conducted using MetaboAnalyst 6.0. Metabolites with *p* < 0.05 and FC > 1.5 or <0.67 were considered significantly altered. Shared differential metabolites (DMs) across comparisons were identified using Venny 2.1 and further analyzed via KEGG pathway enrichment. Pearson correlation analysis of QC samples confirmed data quality (Supplementary Figure S1).

### 2.10 Western blotting analysis

Proteins were isolated from liver tissues and measured using BCA method. After normalization of protein levels with deionized water and the addition of 5× sample buffer, the mixtures were heat-treated at 95°C for 10 min to achieve denaturation. Equivalent protein quantities were loaded onto 10% SDS-PAGE gels for separation, followed by electrotransfer onto PVDF membranes. Membranes were first treated with primary antibodies before being probed with horseradish peroxidase-labeled anti-rabbit secondary antibodies. Protein signals were detected and captured with a Bio-Rad imaging platform (Bio-Rad, United States). Band intensity was quantitatively analyzed using ImageJ software.

### 2.11 Quantitative analysis of metabolites

Quantitative analysis of inosine monophosphate (IMP), adenine, hypoxanthine, adenosine, N^6^-methyladenosine (m6A), and adenosine monophosphate (AMP) in mouse liver tissues was performed by the external standard calibration method. The chromatographic and MS parameters agreed with those utilized in the metabolomics analysis. Details of the standard curves and scanning parameters are provided in Supplementary Tables S2-S3.

### 2.12 Quantification of hepatic cytokines

Liver concentrations of TNF-α, IL-1β, IL-6, and TGF-β1 were determined using ELISA kits, strictly following the manufacturers’ recommended protocols.

### 2.13 Statistical analysis

Data were presented as mean ± SEM. Statistical comparisons across groups were made using one-way ANOVA, followed by Tukey’s *post hoc* test. Analyses were conducted using GraphPad Prism 8.0, and p-values less than 0.05 were deemed statistically significant.

## 3 Results

### 3.1 Chemical profiling of ILW

**FIGURE 2:**
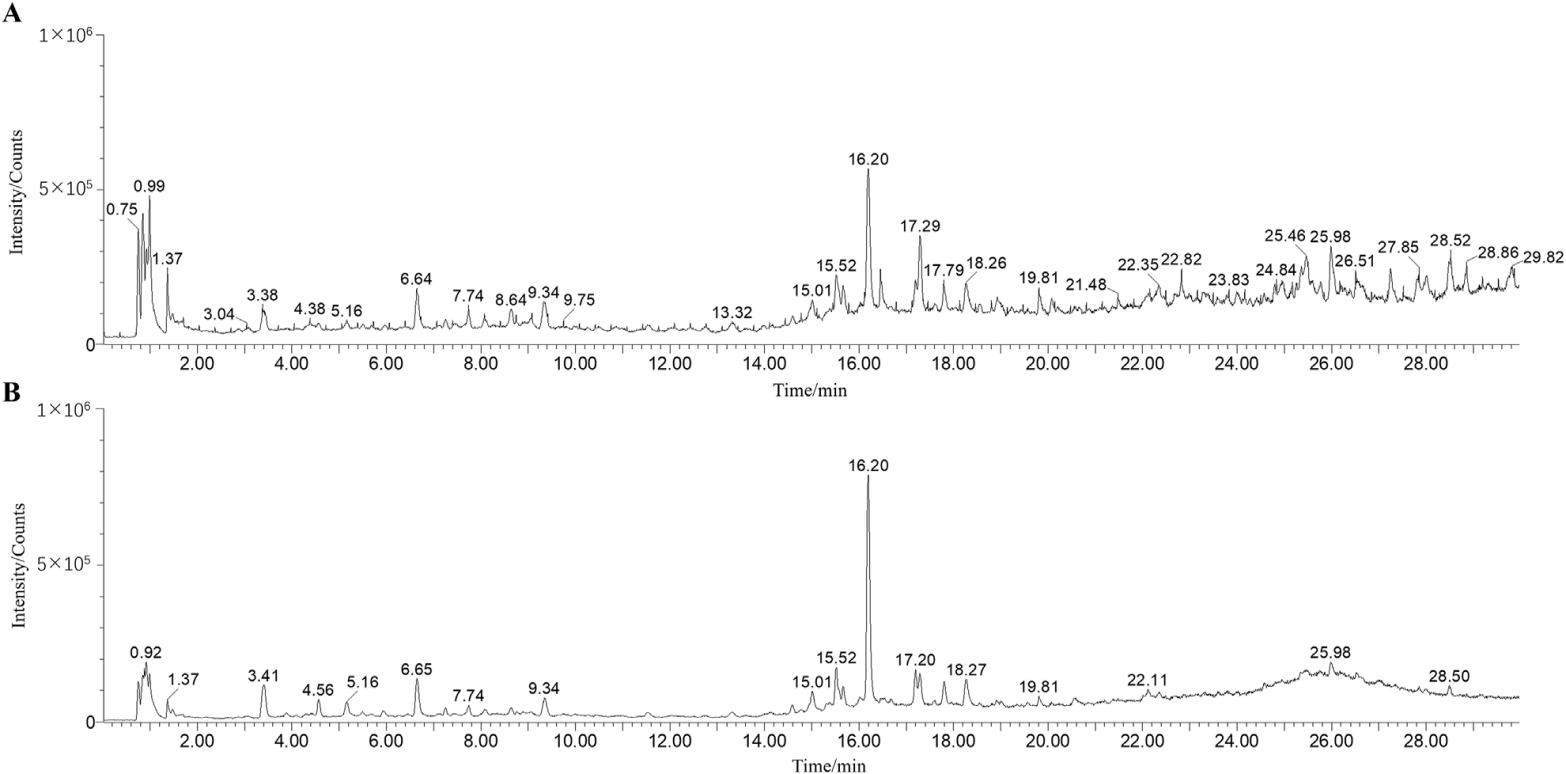
Total ion chromatogram of ILW in positive **(A)** and negative ion **(B)** modes.

A comprehensive chemical analysis of ILW was conducted using UPLC-Q-TOF/MS in positive and negative modes (Figure 2). A total of 32 metabolites were identified, including 9 flavonoids, 6 terpenoids, 14 organic acids, and 3 other metabolites. Among these, 7 metabolites were unequivocally confirmed through comparison with authentic reference standards. An additional 8 metabolites were annotated based on database searches, including PubChem (https://pubchem.ncbi.nlm.nih.gov/), MassBank (https://massbank.eu/MassBank/), and HMDB (https://www.hmdb.ca/). The remaining 17 metabolites were tentatively identified by comparison with previously published literature. Detailed information on all identified metabolites in ILW is summarized in Supplementary Table S4. For instance, metabolite F1 showed a [M + H]^+^ ion at *m/z* 593.150 8, consistent with the molecular formula C_27_H_30_O_15_. Sequential neutral losses of C_3_H_6_O_3_ and C_4_H_8_O_4_ yielded fragment ions at *m/z* 503.120 5 and 473.107 4, respectively. Subsequent loss of either C_3_H_6_O_3_ or C_4_H_8_O_4_ from *m/z* 473.107 4 produced ions at *m/z* 383.074 0 and 353.065 9. This fragmentation behavior, involving characteristic cross-ring cleavages of sugar moieties, is typical of flavone C-glycosides (Guo et al., 2022). Based on MS/MS data, literature comparisons (Picariello et al., 2025), and a reference standard, F1 was identified as vicenin-2. The proposed fragmentation pathway is illustrated in Supplementary Figure S2. In addition, the UPLC chromatograms of the extracts and reference standard of the powdered plant material at three wavelengths are listed in Supplementary Figure S3.

### 3.2 Quantification and UPLC fingerprint analysis

The chromatographic profiles of ILW and corresponding reference standards are presented in Supplementary Figure S4, with calibration curves provided in Supplementary Table S5. The amounts of the following main metabolites in ILW were ascertained by quantitative analysis: caffeic acid (1.10 mg g^-1^), schaftoside (0.12 mg g^-1^), isoschaftoside (0.18 mg g^-1^), and rosmarinic acid (5.13 mg g^-1^). Furthermore, reproducibility was assessed across 12 batches of ILW samples (Supplementary Figure S5). The high similarity values (>0.95), as detailed in Supplementary Tables S6 and S7, demonstrate good batch-to-batch consistency and support the reliability and robustness of the ILW preparation process.

### 3.3 ILW attenuates serum ALT and AST elevations

**FIGURE 3:**
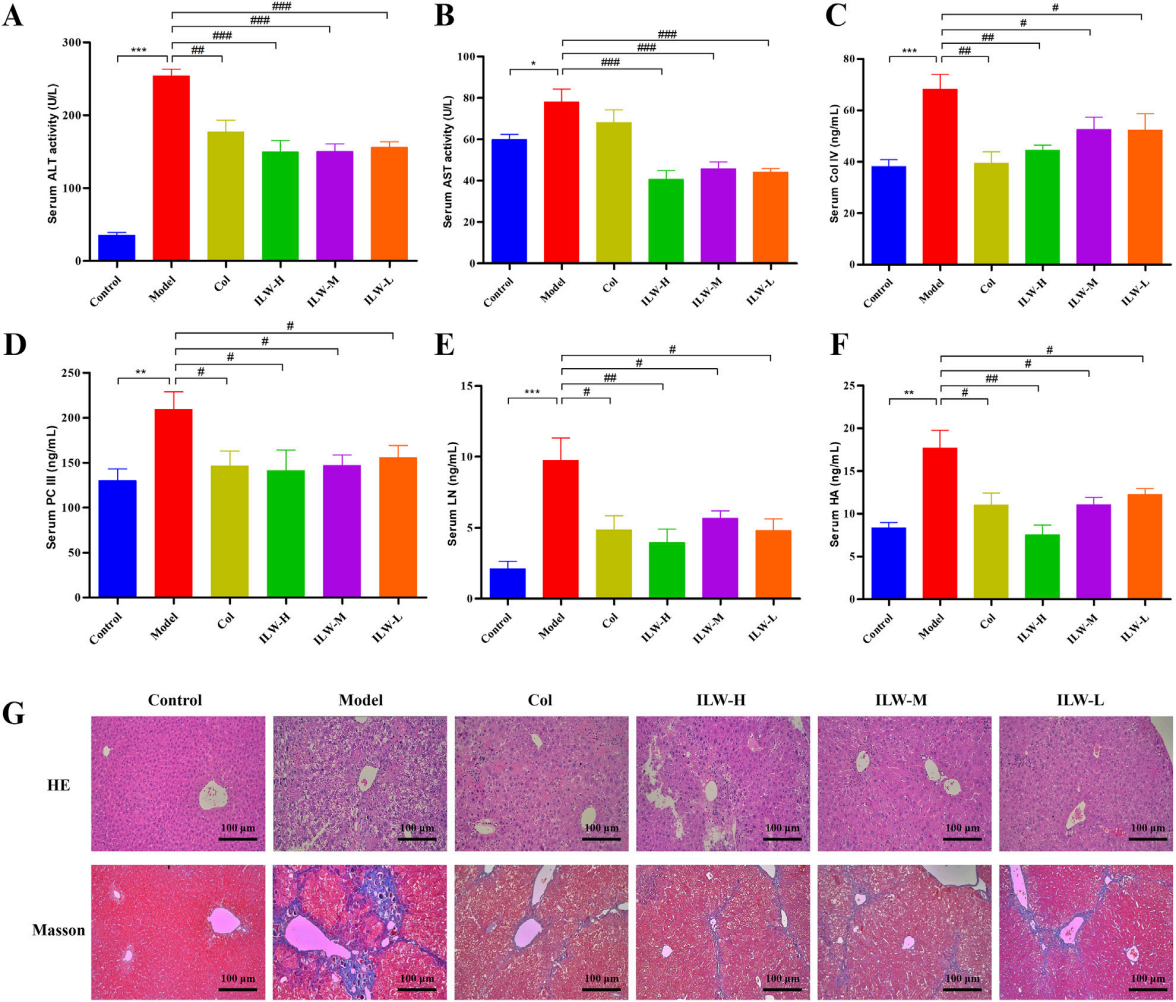
The effect of ILW on mice with liver fibrosis; Effects of ILW on serum ALT **(A)**, AST **(B)**, COL-Ⅳ **(C)**, PC-Ⅲ **(D)**, LN **(E)**, and HA **(F)** in mice with liver fibrosis; **(G)** HE and Masson staining of mouse liver tissue pathological sections. Data presented as mean ± SEM; ^*^ *p* < 0.05, ^**^ *p* < 0.01, ^***^ *p* < 0.001, compared to Control group; ^#^ *p* < 0.05, ^##^ *p* < 0.01, ^###^ *p* < 0.001, compared to Model group.

ALT and AST are widely recognized indicators of liver damage. As shown in Figures 3A,B, serum levels of both ALT and AST were significantly elevated in the model group compared with the control group (*p* < 0.01 or *p* < 0.05), reflecting substantial liver damage induced by fibrosis. Remarkably, ILW administration resulted in a significant reduction in these enzyme levels, with the most pronounced effects observed in the high-dose group (*p* < 0.001), suggesting its potential to alleviate liver injury and restore hepatic function.

### 3.4 ILW reduces serum markers of liver fibrosis

Key indicators of hepatic fibrosis, including serum levels of PC-III, COL-IV, LN, and HA, were markedly higher in the model group than in the control (*p* < 0.001 or *p* < 0.01), as illustrated in Figures 3C–F. ILW treatment significantly reduced their levels across all dosage groups (ILW-H, ILW-M, and ILW-L) (*p* < 0.01 or *p* < 0.05). Notably, ILW-H group exhibited the most pronounced reductions, particularly in PC-III, LN, and HA, surpassing the efficacy of colchicine. These findings highlight ILW’s potential antifibrotic activity and its capacity to mitigate liver fibrosis progression.

### 3.5 ILW attenuates hepatic pathological damage and fibrosis

Histological examination through HE staining revealed extensive hepatocellular disorganization, inflammatory cell infiltration, fibrous tissue proliferation, and architectural disruption of hepatic lobules in the model group (Figure 3G). Colchicine treatment partially restored hepatocyte morphology, improving cellular arrangement and reducing inflammation. Similarly, ILW treatment (high, medium, and low doses) alleviated hepatic injury to varying degrees, progressively restoring normal hepatocyte structure (Figure 3G). Masson staining further confirmed extensive collagen fiber accumulation and fibrous septa formation in the model group, indicating severe hepatic fibrosis. In contrast, colchicine treatment preserved liver architecture with reduced collagen deposition. ILW administration markedly diminished fibrosis, as evidenced by decreased collagen accumulation and improved hepatic lobule integrity across all treatment groups (Figure 3G). These findings highlight ILW’s potential in protecting hepatocytes and mitigating liver fibrosis.

### 3.6 Transcriptomic analysis

**FIGURE 4:**
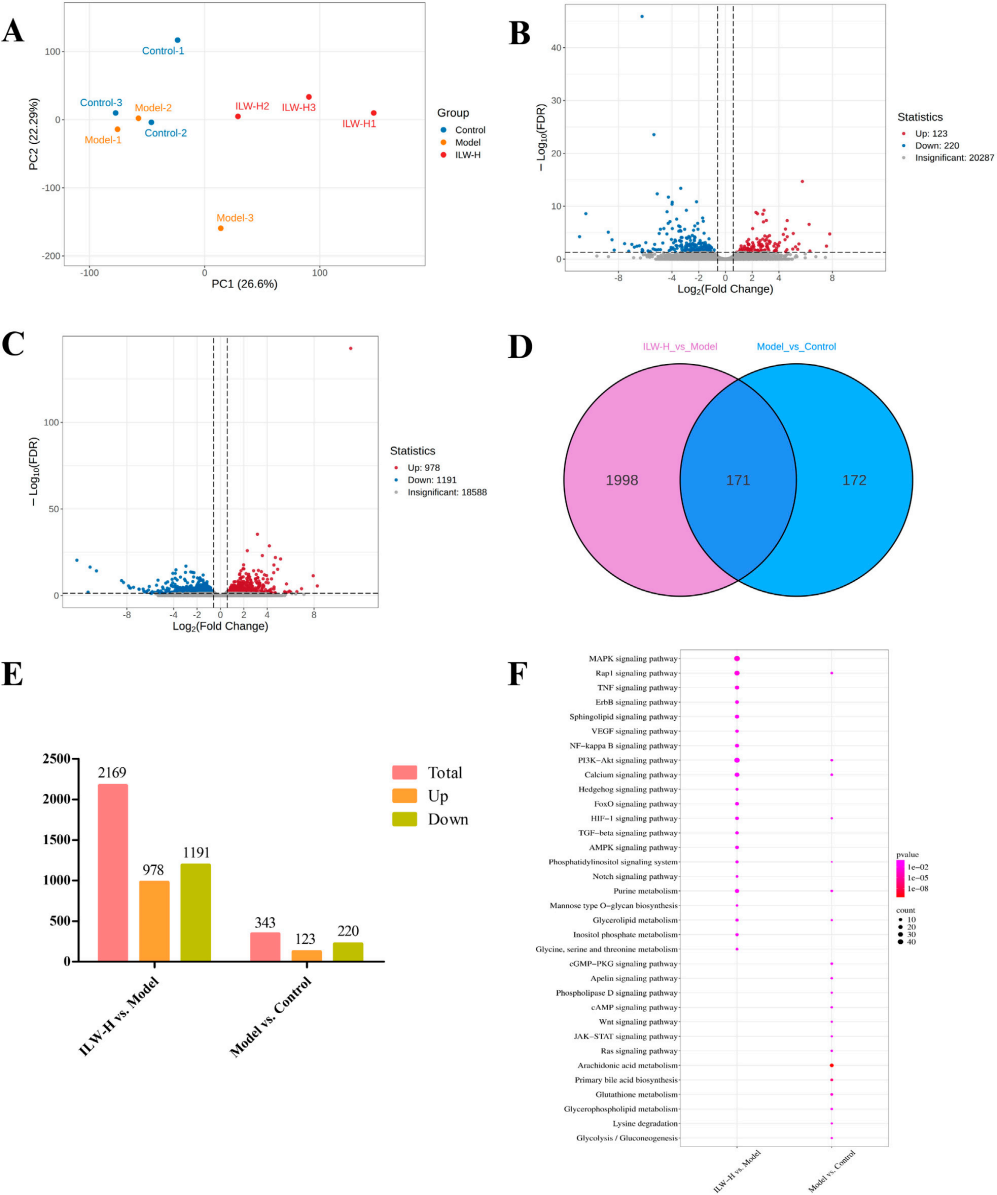
The analysis of DEGs and dysregulated pathways in liver fibrosis by transcriptome experiments. **(A)** PCA score plot of Control, Model and ILW-H groups. **(B)** The volcano plots of DEGs in Model vs. Control group. **(C)** The volcano plots of DEGs in ILW-H vs. Model group. **(D)** Overlaps between DEGs of the two groups. **(E)** The distributions of DEGs for two groups. **(F)** Pathways enriched with DEGs in Model vs. Control group and ILW-H vs. Model group.

The liver, as the primary target of liver fibrosis, undergoes extensive gene expression alterations during disease progression. To explore ILW’s regulatory effects, transcriptome sequencing was conducted on liver tissues. PCA revealed distinct clustering among the groups, with ILW-H partially reversing CCl_4_-induced gene expression changes along the Y-axis (Figure 4A). The two principal components accounted for 26.60% and 22.29% of the variance. A total of 2,341 differentially expressed genes (DEGs) were screened between the Model and Control groups, together with between the ILW-H and Model groups, based on volcano plot analysis using thresholds of FDR <0.05 and FC > 1.5 or <0.67 (Figures 4B,C; Supplementary Figure S6). Specifically, 343 DEGs (123 upregulated, 220 downregulated) distinguished the model from the control group, while ILW-H treatment resulted in 2,169 DEGs (978 upregulated, 1,191 downregulated) in the ILW-H vs. Model comparison (Figures 4D,E). Further analysis identified 171 overlapping DEGs between the two comparisons (Supplementary Table S8). KEGG pathway enrichment analysis highlighted several pathways implicated in disease progression and ILW-mediated therapeutic effects. Seven pathways were enriched in both comparisons (Figure 4F), including those related to inflammation and immunity (Rap1 signaling), metabolism (purine metabolism, glycerolipid metabolism, HIF-1 signaling), and cell proliferation and apoptosis (PI3K-Akt, calcium, and phosphatidylinositol signaling pathways).

### 3.7 Proteomic analysis

**FIGURE 5:**
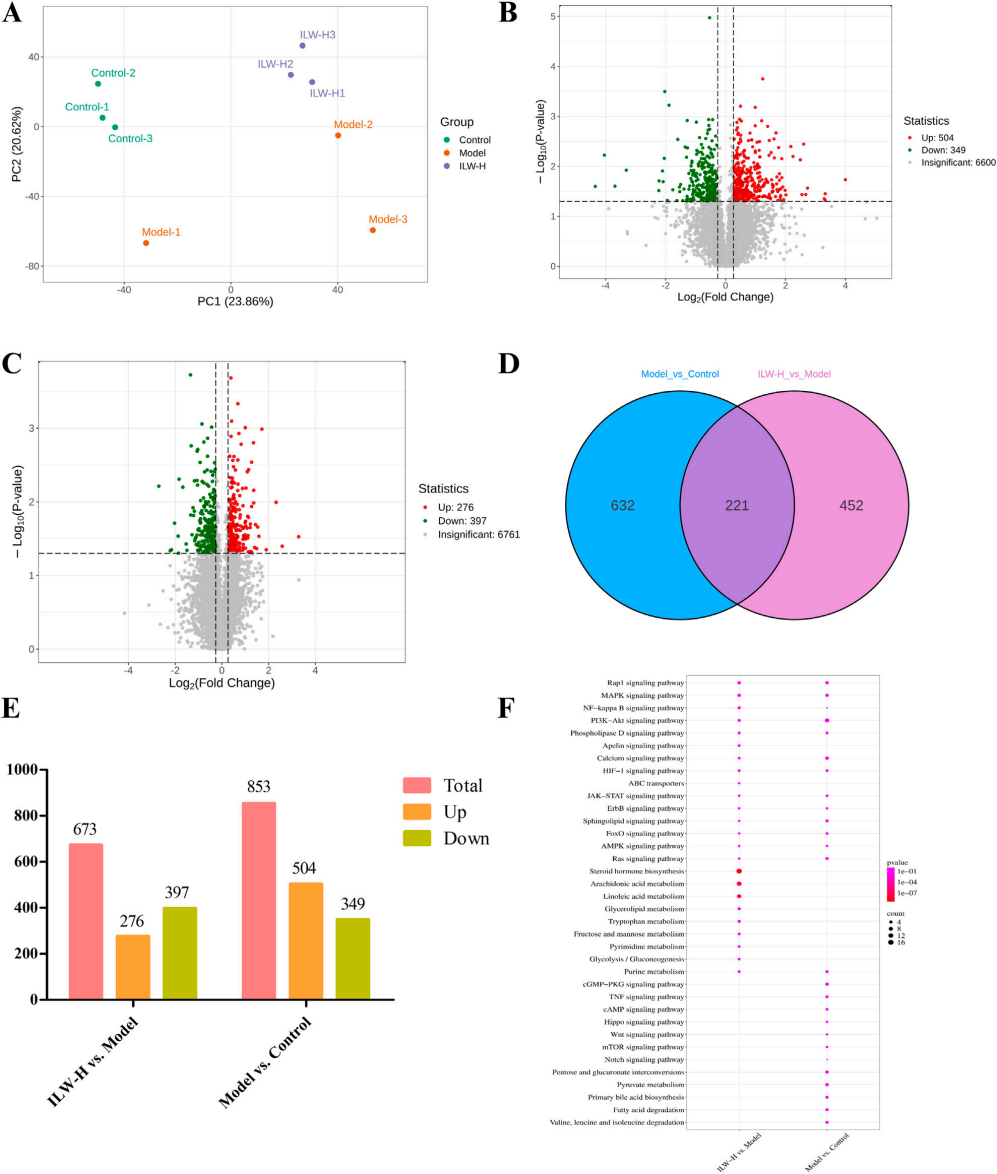
The analysis of DEPs and dysregulated pathways in liver fibrosis by proteomics experiments. **(A)** PCA score plot of Control, Model and ILW-H groups. **(B)** The volcano plots of DEPs in Model vs. Control group. **(C)** The volcano plots of DEPs in ILW-H vs. Model group. **(D)** Overlaps between DEPs of the two groups. **(E)** The distributions of DEPs for two groups. **(F)** Pathways enriched with DEPs in Model vs. Control group and ILW-H vs. Model group.

To investigate protein expression changes associated with liver fibrosis and ILW intervention, label-free proteomic profiling was performed on liver tissues utilizing UHPLC-Orbitrap Astral MS. PCA analysis (Figure 5A) revealed distinct clustering among groups, indicating significant proteomic differences, while intra-group samples remained tightly clustered. DEPs were identified based on a threshold of *p* < 0.05 and FC > 1.2 or <0.83. A total of 1,305 DEPs were detected across comparisons involving the Model group versus the Control and ILW-H groups (Figures 5B,C; Supplementary Figure S7). Specifically, 853 DEPs (504 upregulated, 349 downregulated) were detected in the Model vs. Control group, while ILW-H treatment resulted in 673 DEPs (276 upregulated, 397 downregulated) in the ILW-H vs. Model group (Figures 5D,E). Further analysis identified 221 overlapping DEPs between the two comparisons (Supplementary Table S9). KEGG pathway enrichment analysis highlighted 14 common pathways (Figure 5F), encompassing inflammation and immunity (Rap1, MAPK, NF-κB, and JAK-STAT pathways), metabolism (purine metabolism, sphingolipid signaling, AMPK, HIF-1 pathways), and cell proliferation and apoptosis (PI3K-Akt, calcium, Ras, FoxO, ErbB, and phospholipase D pathways).

### 3.8 Metabolomics analysis

**FIGURE 6:**
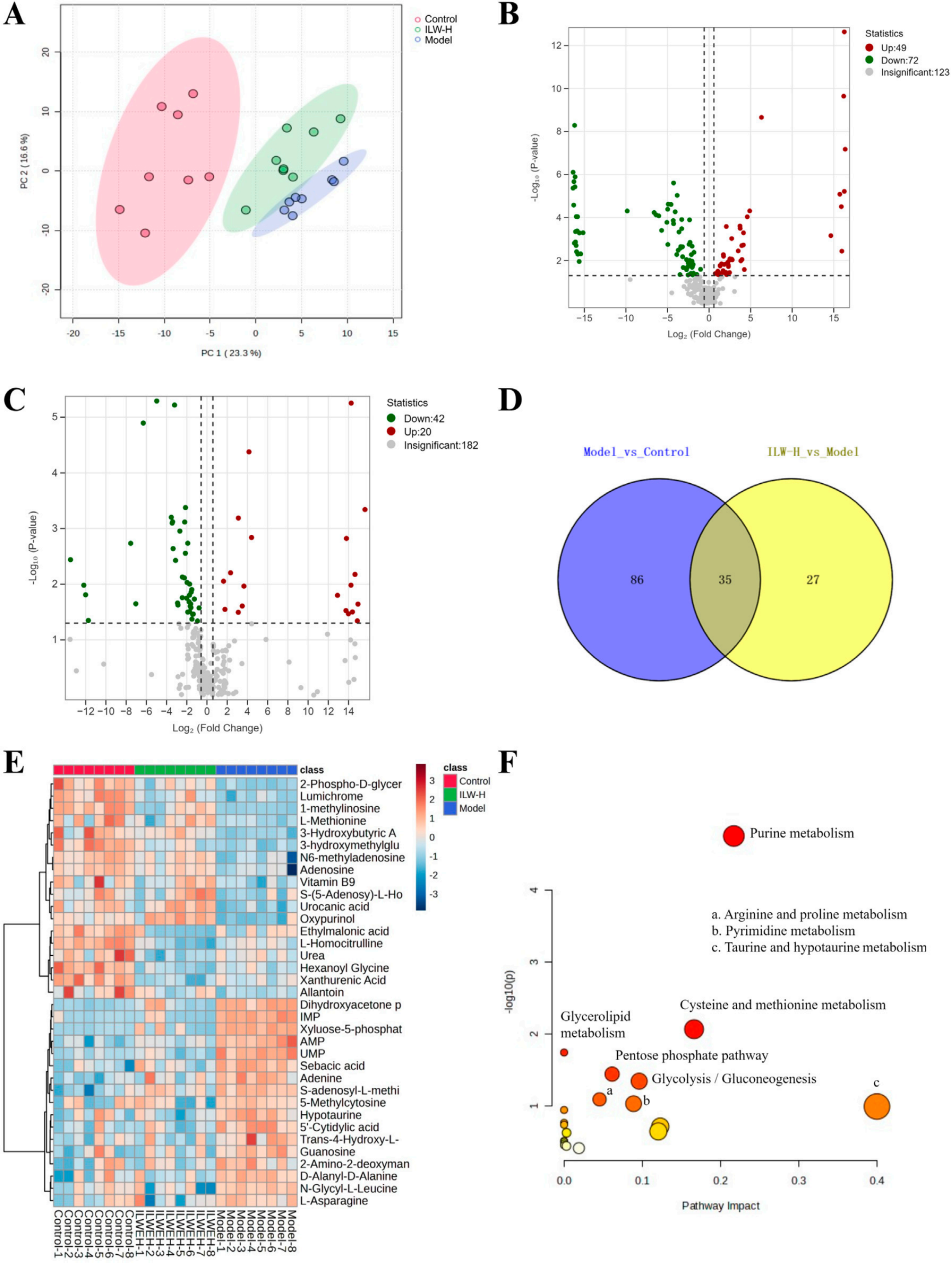
The analysis of DMs and dysregulated pathways in liver fibrosis by metabolomics experiments. **(A)** PCA score plot of Control, Model and ILW-H groups. **(B)** The volcano plots of DMs in Model vs. Control group. **(C)** The volcano plots of DMs in ILW-H vs. Model group. **(D)** Overlaps between DMs of the two groups. **(E)** Heatmap diagram of the shared DMs. **(F)** KEGG metabolic pathways enriched with the intersection DMs in Model vs. Control group and ILW-H vs. Model group.

Comprehensive metabolic profiling of hepatic tissues from the control, model, and ILW-H-treated groups was performed using UPLC-MS/MS, resulting in the detection of 244 metabolites. PCA analysis (Figure 6A) revealed clear separation of the samples into three distinct clusters: the control, model, and ILW-H groups. This indicated significant differences between CCl_4_-induced liver fibrosis and the control, while the ILW-H group showed a partial restorative effect. DMs were identified based on volcano plot criteria (*p* < 0.05, FC > 1.5 or <0.67). A total of 121 DMs were detected between the model and control groups, including 49 upregulated and 72 downregulated metabolites. In comparison, ILW-H treatment resulted in 62 DMs relative to the model group, with 20 upregulated and 42 downregulated (Figures 6B,C). Venn diagram analysis revealed 35 overlapping DMs between the two comparisons (Figure 6D; Table 1), and their relative abundance is visualized in a heatmap (Figure 6E). Pathway enrichment analysis using the MetaboAnalyst platform identified five significant metabolic pathways (*p* < 0.05), including purine metabolism, cysteine and methionine metabolism, glycerolipid metabolism, pentose phosphate pathway, and glycolysis/gluconeogenesis (Figure 6F).

**TABLE 1:** Detailed information on the DMs shared between Model vs. Control and ILW-H vs. Model comparisons.

Name	RT/min	Formula	Precursor ion	Product ion	Ionization model	Model vs. Control			ILW-H vs. Model			Metabolite ID
						*P*-value	Fold change	Trend	*P*-value	Fold change	Trend	
Xyluose-5-phosphate	5.28	C_5_H_11_O_8_P	229.2	97.0	[M-H]^−^	0.0000	78,924.0000	Up	0.0004	0.2288	Up	HMDB0000868
Dihydroxyacetone phosphate	5.11	C_3_H_7_O_6_P	169.0	96.8	[M-H]^−^	0.0000	74,999.0000	Up	0.0018	0.2672	Down	HMDB0001473
IMP	5.76	C_10_H_13_N_4_O_8_P	346.9	78.9	[M-H]^−^	0.0000	79.0870	Up	0.0000	0.1081	Down	HMDB0000175
1-methylinosine	1.31	C_11_H_14_N_4_O_5_	283.1	151.0	[M + H]^+^	0.0000	0.0000	Down	0.0104	19,404.0000	Up	HMDB0002721
UMP	5.40	C_9_H_13_N_2_O_9_P	323.0	96.9	[M-H]^−^	0.0000	84,893.0000	Up	0.0000	0.0318	Down	HMDB0000288
2-Phospho-D-glyceric acid	0.81	C_3_H_7_O_7_P	184.9	78.8	[M-H]^−^	0.0000	0.0000	Down	0.0015	14,227.0000	Up	HMDB0003391
3-hydroxymethylglutaric acid	1.57	C_6_H_10_O_5_	161.1	57.0	[M-H]^−^	0.0000	0.0000	Down	0.0159	7,699.8000	Up	HMDB0000355
L-Homocitrulline	5.83	C_7_H_15_N_3_O_3_	190.1	84.1	[M + H]^+^	0.0000	0.0519	Down	0.0155	0.0002	Down	HMDB0000679
Lumichrome	3.94	C_12_H_10_N_4_O_2_	241.1	198.0	[M-H]^−^	0.0000	0.0000	Down	0.0298	13,759.0000	Up	HMDB0254199
AMP	5.38	C_10_H_14_N_5_O_7_P	345.9	79.0	[M-H]^−^	0.0000	78,985.0000	Up	0.0000	0.0127	Down	HMDB0000045
Hexanoyl Glycine	3.68	C_8_H_15_NO_3_	174.1	99.0	[M + H]^+^	0.0000	0.0419	Down	0.0449	0.0003	Down	HMDB0000701
Adenine	1.37	C_5_H_5_N_5_	134.0	107.0	[M-H]^−^	0.0000	61,602.0000	Up	0.0347	0.3735	Down	HMDB0000034
S-adenosyl-L-methioninate	8.72	C_15_H_22_N_6_O_5_S	399.1	136.1	[M + H]^+^	0.0000	29.6160	Up	0.0185	0.4201	Down	HMDB0000988
Xanthurenic Acid	2.46	C_10_H_7_NO_4_	204.1	160.0	[M-H]^−^	0.0001	0.0592	Down	0.0104	0.0002	Down	HMDB0000881
Sebacic acid	0.75	C_10_H_18_O_4_	201.1	201.1	[M-H]^−^	0.0001	24.1610	Up	0.0028	0.2272	Down	HMDB0000792
Oxypurinol	1.30	C_5_H_4_N_4_O_2_	153.0	136.0	[M + H]^+^	0.0001	0.1967	Down	0.0000	17.7940	Up	HMDB0000786
3-Hydroxybutyric Acid	1.46	C_4_H_8_O_3_	103.1	59.0	[M-H]^−^	0.0004	0.0000	Down	0.0000	19,677.0000	Up	HMDB0000011
Urocanic acid	1.23	C_6_H_6_N_2_O_2_	139.0	121.0	[M + H]^+^	0.0005	0.0000	Down	0.0005	50,713.0000	Up	HMDB0000301
N6-methyladenosine	1.22	C_11_H_15_N_5_O_4_	282.1	150.1	[M + H]^+^	0.0005	0.0000	Down	0.0067	25,582.0000	Up	HMDB0004044
5′-Cytidylic acid	6.24	C_9_H_14_N_3_O_8_P	324.05	112.0	[M + H]^+^	0.0009	6.6001	Up	0.0011	0.1556	Down	HMDB0000095
Allantoin	1.63	C_4_H_6_N_4_O_3_	157.04	97.01	[M-H]^−^	0.0039	0.0000	Down	0.0340	16,267.0000	Up	HMDB0000462
Ethylmalonic acid	2.30	C_5_H_8_O_4_	131.0	87.1	[M-H]^−^	0.0042	0.2844	Down	0.0344	0.3887	Down	HMDB0000622
L-Methionine	1.23	C_5_H_11_NO_2_S	150.05	104.0	[M + H]^+^	0.0048	0.0000	Down	0.0456	30,009.0000	Down	HMDB0000696
Adenosine	1.43	C_10_H_13_N_5_O_4_	268.1	136.1	[M + H]^+^	0.0049	0.0000	Down	0.0314	21,447.0000	Up	HMDB0000050
N-Glycyl-L-Leucine	1.73	C_8_H_16_N_2_O_3_	189.1	86.0	[M + H]^+^	0.0053	3.2989	Up	0.0139	0.3375	Down	HMDB0000759
Trans-4-Hydroxy-L-Proline	4.80	C_5_H_9_NO_3_	132.1	86.0	[M + H]^+^	0.0090	6.1114	Up	0.0157	0.3008	Down	HMDB0000725
S-(5-Adenosy)-L-Homocysteine	1.23	C_14_H_20_N_6_O_5_S	385.0	136.1	[M + H]^+^	0.0098	0.2008	Down	0.0320	8.5472	Up	HMDB0000939
Vitamin B9	2.60	C_19_H_19_N_7_O_6_	442.2	295.1	[M + H]^+^	0.0111	0.0000	Down	0.0227	31,354.0000	Up	HMDB0000121
D-Alanyl-D-Alanine	2.60	C_6_H_12_N_2_O_3_	161.09	44.04	[M + H]^+^	0.0141	2.8326	Up	0.0008	0.2166	Down	HMDB0003459
Hypotaurine	4.58	C_2_H_7_NO_2_S	110.0	92.0	[M + H]^+^	0.0169	2.5023	Up	0.0008	0.0945	Down	HMDB0000965
Guanosine	2.62	C_10_H_13_N_5_O_5_	282.0	150.0	[M-H]^−^	0.0179	2.5240	Up	0.0125	0.3515	Down	HMDB0000133
L-Asparagine	5.48	C_4_H_8_N_2_O_3_	131.0	114.0	[M-H]^−^	0.0312	2.0775	Up	0.0424	0.3558	Down	HMDB0000168
5-Methylcytosine	8.50	C_5_H_7_N_3_O	126.1	109.0	[M + H]^+^	0.0400	1.6793	Up	0.0458	0.5214	Down	HMDB0002894
2-Amino-2-deoxymannose	4.53	C_6_H_13_NO_5_	180.1	72.1	[M + H]^+^	0.0421	2.1102	Up	0.0178	0.2489	Down	——
Urea	1.19	CH_4_N_2_O	61.0	44.0	[M + H]^+^	0.0466	0.1739	Down	0.0036	0.0001	Down	HMDB0000294

### 3.9 Integrated multi-omics analysis

**FIGURE 7:**
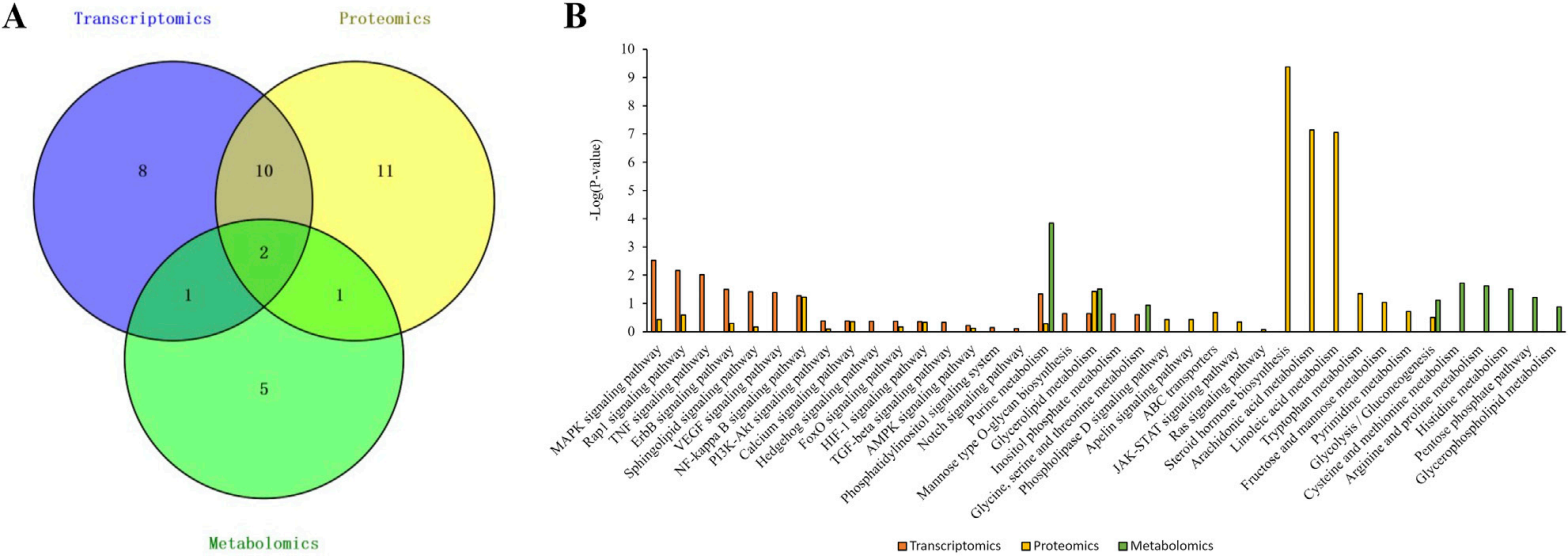
Integrated analysis of transcriptomics, proteomics, and metabolomics. **(A)** Venn diagram. **(B)** Bar graph of the KEGG pathway enrichment analysis results.

To uncover central molecular mechanisms underlying ILW’s antifibrotic action, an integrated analysis of transcriptomic, proteomic, and metabolomic datasets was performed. Cross-omics comparison identified 12 enriched pathways shared between transcriptomics and proteomics, 3 overlapping between transcriptomics and metabolomics, and another 3 common to proteomics and metabolomics (Figure 7A). Notably, purine metabolism and glycerolipid metabolism were consistently enriched across all three omics layers (Figure 7B). Purine metabolism plays an essential role in maintaining intracellular energy homeostasis, regulating inflammatory signaling cascades, and modulating the activation of hepatic stellate cells (HSCs). In parallel, glycerolipid metabolism is integral to lipid equilibrium and membrane remodeling. In the context of CCl_4_-induced liver fibrosis, purine metabolism emerges as particularly relevant, as its intermediates—most notably adenosine—function as key bioactive molecules capable of modulating the NF-κB signaling pathway. This pathway, consistently identified in both transcriptomic and proteomic analyses, serves as a central node in the pathogenesis of liver fibrosis, orchestrating inflammatory responses, immune cell recruitment, HSC activation, and apoptotic processes. These findings collectively underscore the purine metabolism and NF-κB signaling as critical pathways for further in-depth mechanistic investigation.

### 3.10 ILW-mediated modulation of the purine metabolism pathway

**FIGURE 8:**
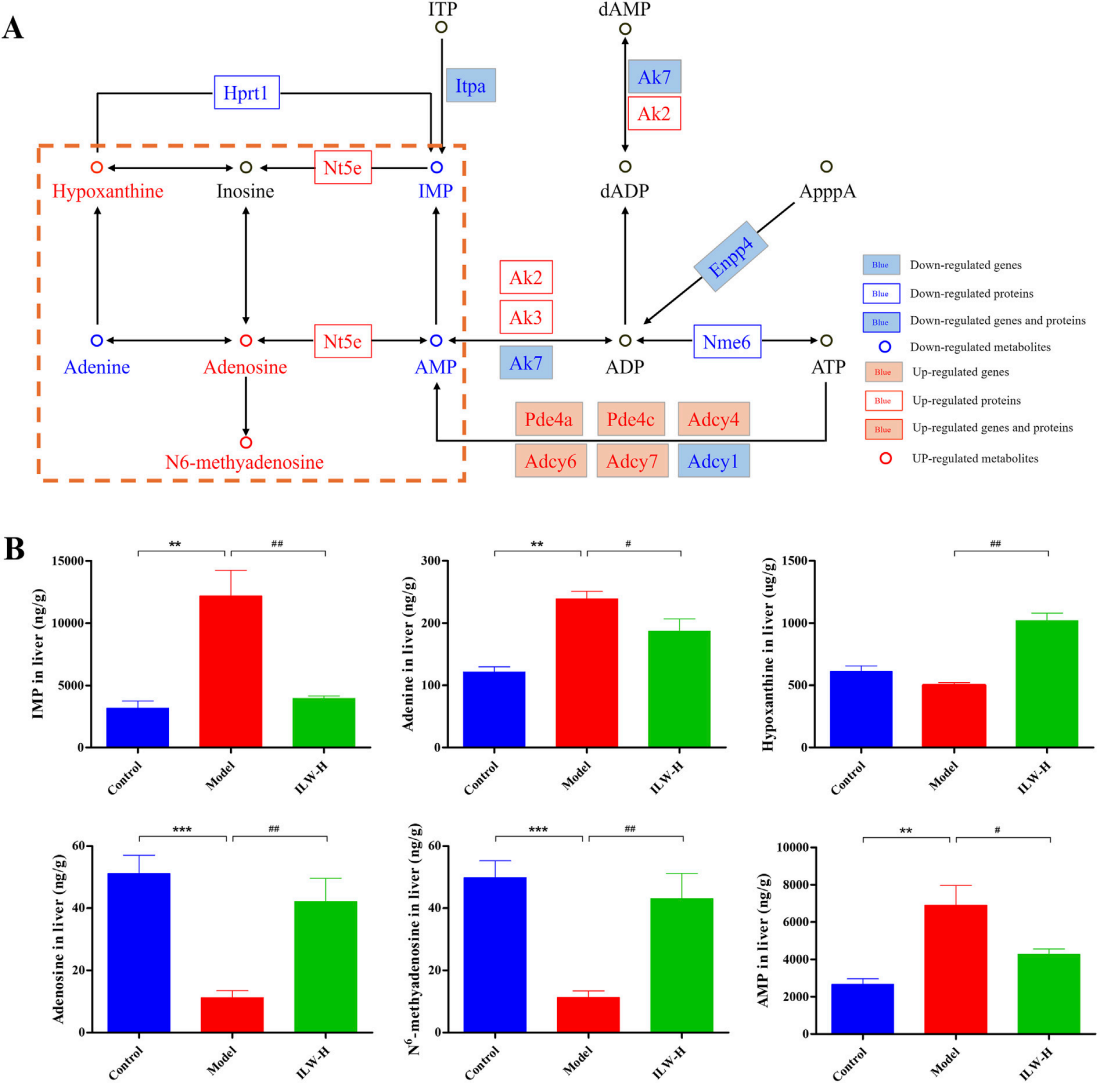
Regulatory effects of ILW on purine metabolism pathway. **(A)** Regulation of ILW on purine metabolism in mice with liver fibrosis. **(B)** Concentrations of IMP, adenine, hypoxanthine, adenosine, N^6^-methyladenosine, and AMP in the Control, Model, and ILW-H groups (n = 8). ^**^ *p* < 0.01, ^***^ *p* < 0.001, compared to Control group; ^#^ *p* < 0.05, ^##^ *p* < 0.01, compared to Model group.

To further elucidate ILW’s influence on purine metabolism, DEGs, DEPs, and DMs were mapped to this pathway. As shown in Figure 8A, ILW-H modulated a total of 9 DEGs, 5 DEPs, and 6 DMs. Specifically, 5 DEGs (Pde4a, Pde4c, Adcy4, Adcy6, and Adcy7) were upregulated, while 4 (Adcy1, Enpp4, Ak7, and Itpa) were downregulated. Additionally, ILW-H treatment increased the expression of 3 DEPs (Nt5e, Ak2, and Ak3) while reducing 2 (Nme6 and Hprt1). Metabolomic profiling further identified 3 upregulated DMs (adenosine, hypoxanthine, and m6A) and 3 downregulated DMs (IMP, AMP, and adenine). Functionally, adenylate cyclase types 1, 4, 6, and 7 (ADCY1, ADCY4, ADCY6, ADCY7) convert adenosine triphosphate (ATP) to cyclic adenosine monophosphate (cAMP), whereas phosphodiesterase 4A and 4C (PDE4A and PDE4C) hydrolyze cAMP into adenosine monophosphate (AMP). Meanwhile, adenylate kinases (AK7, AK2, and AK3) facilitate the exchange of phosphate groups between ATP and AMP, maintaining the balance of ATP, AMP and adenosine diphosphate (ADP). Besides, Hprt1 and Itpa are involved in the synthesis of inosine monophosphate (IMP).

Based on metabolomics results, we further analyzed seven purine metabolites, including IMP, AMP, adenine, adenosine, hypoxanthine, m6A, and inosine. These are key intermediates in both degradation and salvage branches of purine metabolism. Inosine was included due to its close connection—it is derived from AMP, IMP, or adenosine, and is a precursor of hypoxanthine. Targeted quantification (Supplementary Tables S2–S3) showed that ILW-H significantly decreased hepatic IMP, AMP, and adenine levels (*p* < 0.05), while increasing adenosine, hypoxanthine, and m6A (*p* < 0.01) (Figure 8B). Inosine showed a rising trend after ILW-H intervention, though the change was not statistically significant (Supplementary Figure S8). Notably, Nt5e protein, which catalyzes AMP dephosphorylation to adenosine, was significantly upregulated in ILW-H group (*p* < 0.05, Supplementary Figure S9). This aligns with the observed elevation in adenosine. Together, these results suggest that ILW exerts a multi-level regulatory effect on purine metabolism in fibrotic liver tissue.

### 3.11 Regulatory effects of ILW on NF-κB pathway

**FIGURE 9:**
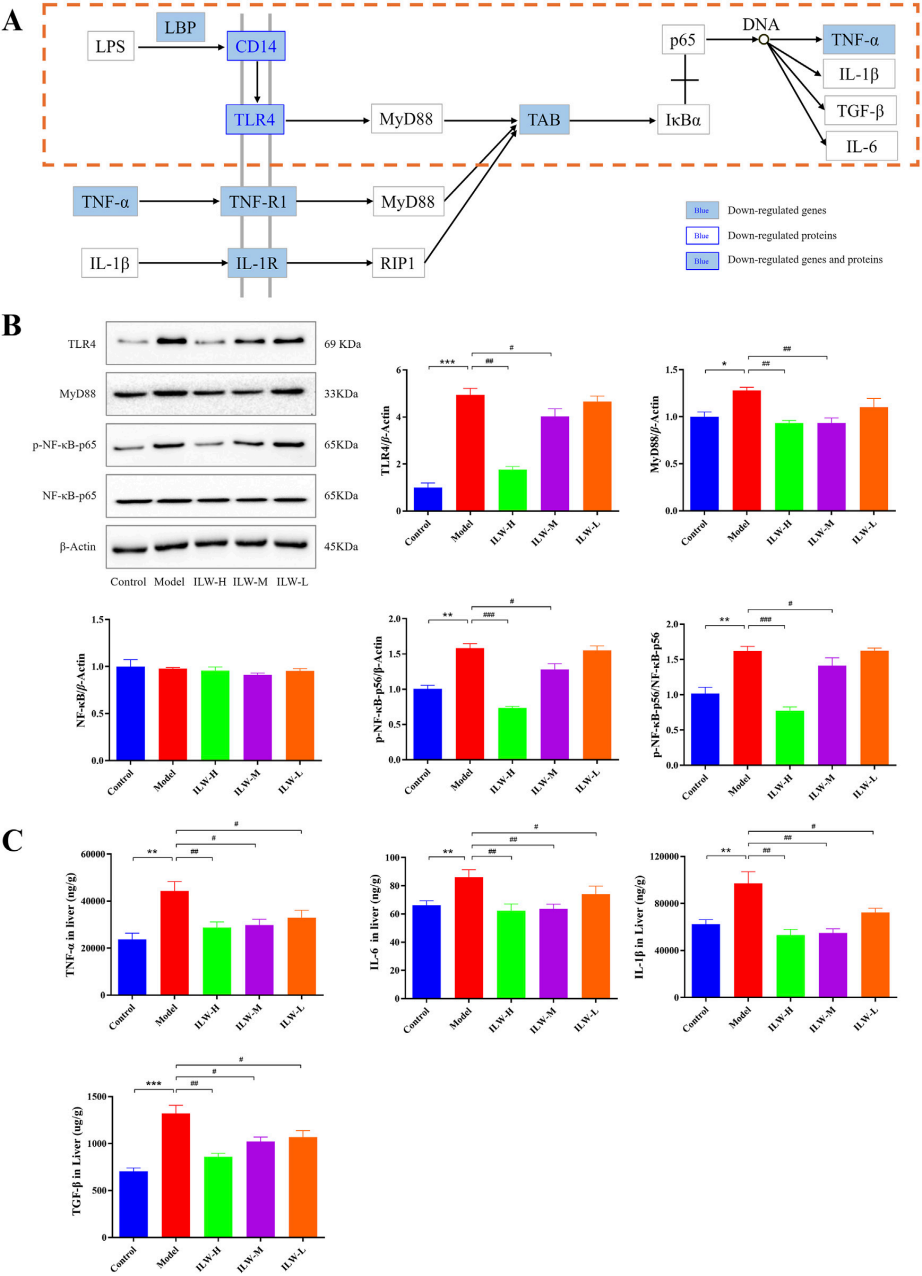
Regulatory effects of ILW on NF-κB signaling pathway. **(A)** Regulation of ILW on NF-κB signaling pathway in mice with liver fibrosis. **(B)** Representative immunoblotting images and quantification of TLR4, MyD88, NF-κB-p56, and p-NF-κB-p56 in protein levels (n = 3). **(C)** Concentrations of TNF-α, IL-6, IL-1β and TGF-β in the Control, Model, and ILW-H groups (n = 8). Data presented as mean ± SEM; ^*^ *p* < 0.05, ^**^ *p* < 0.01, ^***^ *p* < 0.001, compared to Control group; ^##^ *p* < 0.01, ^###^ *p* < 0.001, compared to Model group.

Similarly, we mapped the data from transcriptomics, proteomics, and metabolomics to the NF-κB pathway (Figure 9A). Relative to the model group, ILW-H treatment led to reduced expression of seven critical genes (LBP, CD14, TLR4, TAB, TNF-α, TNF-R1, and IL-1R) as well as two key proteins, CD14 and TLR4, suggesting a suppressive effect on the NF-κB pathway. Considering the pivotal role of TLR4, MyD88, and NF-κB in mediating inflammatory responses, their protein expression levels were further evaluated by Western blot analysis. As shown in Figure 9B, the model group exhibited significantly increased expression of TLR4, MyD88, phosphorylated NF-κB p65 (*p*-NF-κB), and the *p*-NF-κB/NF-κB ratio compared with the control group (*p* < 0.05, *p* < 0.01, or *p* < 0.001). These increases were attenuated by ILW treatment, particularly at medium (ILW-M) and high doses (ILW-H) (*p* < 0.05, *p* < 0.01, or *p* < 0.001), indicating effective suppression of NF-κB activation. Moreover, downstream cytokines regulated by NF-κB signaling, including TNF-α, IL-1β, TGF-β1, and IL-6, were quantitatively assessed in liver tissues via ELISA (Figure 9C). The model group showed significantly elevated levels of these cytokines relative to the control group (*p* < 0.01 or *p* < 0.001). ILW treatment significantly reduced hepatic concentrations of TNF-α (*p* < 0.05 or *p* < 0.01), IL-6 (*p* < 0.05 or *p* < 0.01), IL-1β (*p* < 0.05 or *p* < 0.01), and TGF-β1 (*p* < 0.05 or *p* < 0.01), especially at medium and high doses. Collectively, these results indicate that ILW mitigates the activation of the TLR4/MyD88/NF-κB signaling pathway and suppresses the production of pro-inflammatory cytokines, thereby exerting protective effects against CCl_4_-induced hepatic inflammation and fibrosis.

## 4 Discussion

Liver fibrosis, characterized by ECM deposition, progressively disrupts hepatic architecture and function, ultimately leading to cirrhosis and hepatic failure (Aydın and Akçalı, 2018). Given the limited efficacy and safety concerns of current antifibrotic therapies, traditional Chinese medicine (TCM) and ethnopharmacology have garnered increasing attention for their multifaceted therapeutic potential (Zhou Q. M. et al., 2024; Li et al., 2025b). *I*. *lophanthoides*, a plant long used in folk medicine and dietary applications, exhibits a wide range of pharmacological activities, including hepatoprotection, anti-inflammatory, antimicrobial, and anticancer effects (Li et al., 2020; Sun et al., 2020). It is also incorporated into health products such as Xihuangcao granules and tea. In this study, we employed an integrative strategy encompassing phytochemical profiling, pharmacological efficacy evaluation, and multi-omics analyses—including transcriptomics, proteomics, and metabolomics—to elucidate the hepatoprotective and antifibrotic mechanisms of ILW.

Liver health is crucial for maintaining metabolic and nutritional balance. The CCl_4_-induced hepatic fibrosis model replicates the pathological features of human liver fibrosis, including hepatocyte injury, inflammatory infiltration, activation of HSCs, and ECM buildup, making it a dependable platform for assessing anti-fibrotic interventions (Xu et al., 2024; Ye et al., 2024). In the present study, ILW significantly lowered serum ALT and AST levels, markers of liver damage. Furthermore, ILW significantly diminished fibrotic progression, as evidenced by reduced serum concentrations of fibrosis-related markers such as PC-III, COL-IV, LN, and HA. Histopathological examination through HE and Masson staining revealed that ILW markedly attenuated liver inflammation, fibrosis, and structural damage. Collectively, these findings imply that ILW exerts hepatoprotective effects by preventing collagen accumulation and preserving liver function.

Multi-omics integration revealed that ILW profoundly modulates signaling networks related to inflammation, metabolism, and cell proliferation. Among these, purine metabolism and NF-κB signaling were consistently enriched across transcriptomic, proteomic, and metabolomic analyses, underscoring their central roles in ILW’s anti-fibrotic mechanism. KEGG enrichment of 35 differential metabolites identified purine metabolism as the most significantly affected pathway. Purine metabolism plays a dual role in maintaining energy balance and regulating immune responses, and its dysregulation has been increasingly linked to the pathogenesis of hepatic fibrosis (Velázquez-Miranda et al., 2019). Perturbations in purine metabolism contribute to inflammatory responses, partly through metabolites such as adenosine, which modulates local immune activity and tissue injury (Haskó and Cronstein, 2013; Wang et al., 2020; Pasquini et al., 2021). Through metabolomics and targeted metabolite quantification, ILW was shown to regulate six key purine intermediates in fibrotic liver tissue: IMP, AMP, adenine, adenosine, hypoxanthine, and m6A. Among them, adenosine is a well-characterized anti-inflammatory molecule with established protective roles in models of liver injury and fibrosis (Hernández-Muñoz et al., 2001; Yang et al., 2024). In parallel, m6A—the most abundant internal mRNA modification—has emerged as a critical epigenetic regulator of immune homeostasis. Notably, METTL3-mediated m6A methylation has been shown to suppress Toll-like receptor (TLR) signaling and downstream inflammatory responses (Geng et al., 2023; Xie et al., 2023). In our study, ILW-H not only altered levels of purine metabolites such as IMP, AMP, adenine, and hypoxanthine—reflecting a broader metabolic reprogramming during fibrogenesis—but also significantly upregulated Nt5e, which catalyzes AMP dephosphorylation to adenosine. This change was consistent with elevated hepatic levels of both adenosine and m6A, providing molecular evidence for ILW’s immunoregulatory and anti-fibrotic efficacy via purine metabolism remodeling. In addition to its metabolic effects, ILW robustly inhibited NF-κB pathway activation. Multi-omics data showed reduced expression of key upstream receptors (TLR4, CD14, TNF-α) and downstream effectors in fibrotic liver. The TLR4–MyD88–NF-κB axis is a well-established driver of hepatic fibrosis, promoting hepatocyte injury, HSC activation, and pro-inflammatory cytokine release (Rodríguez et al., 2021; Wu et al., 2024). Upon stimulation, Kupffer cells—enriched in TLR4 and NOD-like receptors—activate the MyD88-dependent cascade, culminating in NF-κB-mediated transcription of IL-1β, TNF-α, and IL-6 (Wang et al., 2023b). These immune cells, along with activated HSCs, also release TGF-β1, a master profibrotic cytokine that drives extracellular matrix deposition and collagen synthesis (Liu et al., 2020). In the present study, ILW-H and ILW-M administration significantly downregulated protein levels of TLR4, MyD88, phosphorylated NF-κB p65, and downstream cytokines including TNF-α, IL-6, IL-1β, and TGF-β1 in the livers of fibrotic mice. Together, these findings highlight a dual regulatory role of ILW—modulating both metabolic (purine) and inflammatory (NF-κB) circuits—to exert anti-fibrotic effects in liver fibrosis.

To elucidate ILW’s bioactive metabolites, we established a rapid UPLC-Q-TOF/MS method that enabled metabolite profiling within 30 min, identifying 32 major metabolites. Further, four key metabolites, including caffeic acid, schaftoside, isoschaftoside, and rosmarinic acid, were quantitatively measured by UPLC. The preliminary serum pharmacochemistry analysis by our research group revealed that caffeic acid, schaftoside, and rosmarinic acid could be absorbed into the bloodstream (Chen K. K. et al., 2024). These metabolites have been previously reported to exhibit hepatoprotective and antifibrotic properties (Yu et al., 2024; Elufioye and Habtemariam, 2019; Reyes et al., 1995; Chen L. et al., 2024; Abe, 2025), and likely contribute synergistically to the therapeutic activity of ILW.

**FIGURE 10:**
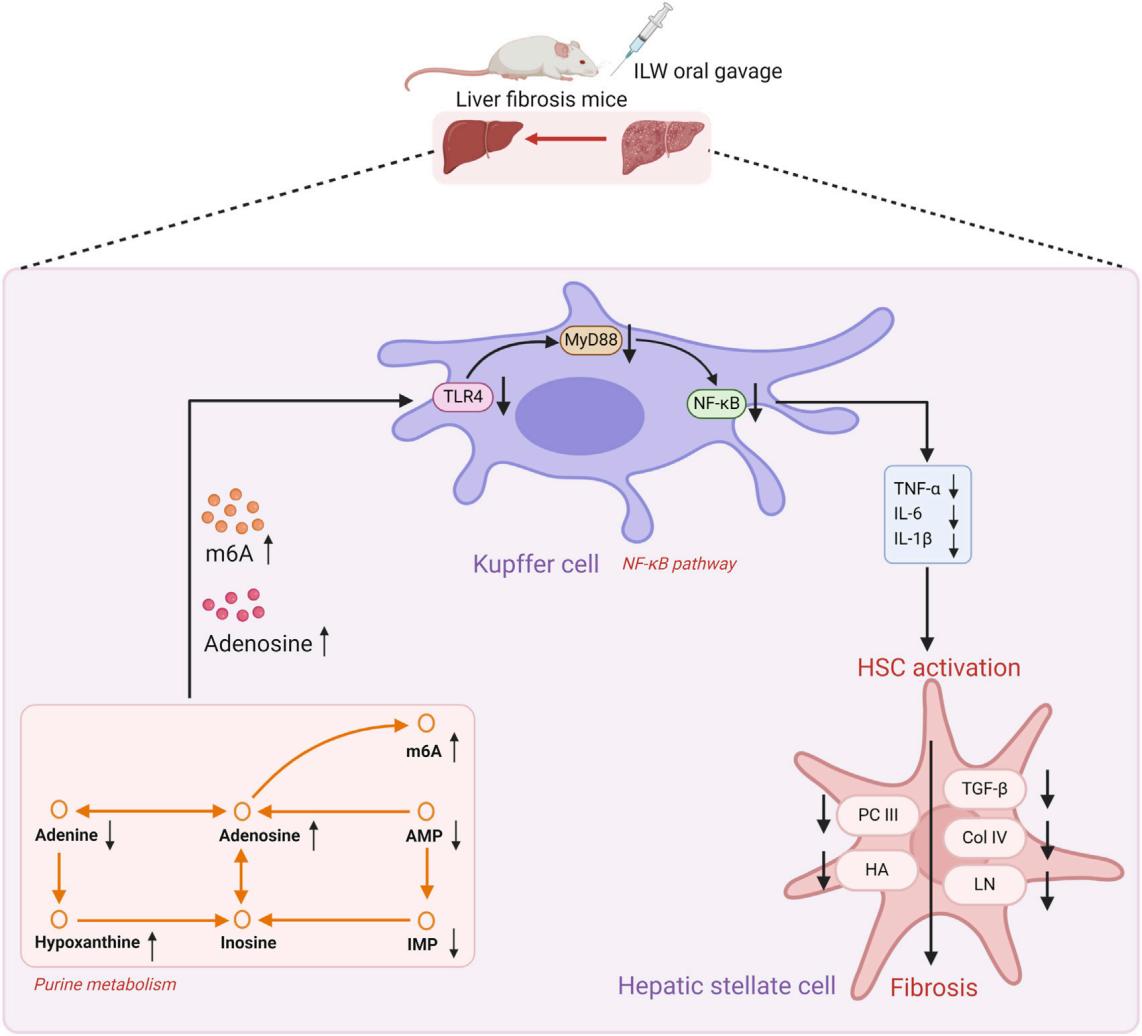
Proposed molecular mechanism underlying the anti-liver fibrosis effects of ILW.

In summary, ILW ameliorates liver fibrosis by modulating purine metabolism and inhibiting NF-κB-driven inflammation, HSC activation, and collagen deposition (Figure 10). These findings provide a mechanistic rationale for the traditional use of *I. lophanthoides* in clearing heat and toxins, eliminating dampness and jaundice, and cooling the blood to resolve stasis. Nevertheless, this study has several limitations. A major limitation is the relatively high dose of ILW-H group used (1.88 g/kg/day). Although body surface area (BSA)-based dose conversion is a common approach for interspecies scaling, it can yield physiologically unrealistic or clinically irrelevant doses when starting concentrations are high (Reagan-Shaw et al., 2008; Xu, 2002). For complex botanical extracts, excessive dosing increases the risk of metabolic overload, off-target effects, toxicity, and experimental artefacts, thereby limiting the interpretability and translational value of the findings (Heinrich et al., 2020). To ensure dose relevance, especially in studies involving high starting doses, BSA conversion should be complemented by pharmacokinetic data, bioavailability, and expected human exposure. Future studies should aim to define the minimum effective dose and validate efficacy at lower, clinically relevant concentrations. Another limitation is the exclusive use of a CCl_4_-induced fibrosis model. While this model is well-established, it does not fully capture the diverse etiologies of liver fibrosis. To strengthen the generalizability of our findings, future investigations should incorporate alternative models, including those based on non-alcoholic steatohepatitis (NASH), cholestasis, and chronic alcohol-induced liver injury. Addressing these limitations will be critical to fully elucidate the anti-fibrotic potential and underlying mechanisms of ILW.

## 5 Conclusion

This study provides a systematic and integrative evaluation of the antifibrotic potential of ILW in a CCl_4_-induced mouse model of liver fibrosis. Through integrated transcriptomics, proteomics, and metabolomics analyses, we identified that ILW exerts its protective effects via modulation of purine metabolism and suppression of NF-κB-mediated inflammatory signaling and collagen deposition. Besides, the major metabolites in ILW were comprehensively elucidated by qualitative and quantitative analysis. Collectively, these findings provide novel mechanistic insights into the anti-fibrotic activity of *I. lophanthoides* and establish a pharmacological basis for its traditional use in liver-related disorders. Moreover, the results offer a scientific rationale for the future development of ILW-based therapeutics targeting liver fibrosis at both metabolic and molecular levels.

## Data Availability

The datasets presented in this study can be found in online repositories. The names of the repository/repositories and accession number(s) can be found in the article/Supplementary Material.
