# Supplementary material for: Isodon lophanthoides alleviates liver fibrosis via modulation of purine metabolism and NF-κB signaling pathway: insights from multi-omics analysis
Source: Front Pharmacol. 2025 Aug 1;16:1630927. doi: 10.3389/fphar.2025.1630927 (PMC12354614; doi:10.3389/fphar.2025.1630927)
Supplement: Supplementary file 1 [file DataSheet1.docx]

Supplementary Material

**1 Determination of index component in ILW**

**1.1** **Preparation of test solution**

Accurately weigh 0.8 g of the *Isodon lophanthoides* water extract (ILW, batch No. 202209013) and place it in a stoppered conical flask. Add 20 mL of methanol, record the total mass, and stopper the flask. Subject the mixture to ultrasonic treatment (power: 99%, frequency: 40 kHz) for 30 minutes. Compensate for any mass loss due to evaporation by adding methanol. Centrifuge the supernatant at 10,000 rpm for 10 minutes and filter it through a 0.22 μm microporous membrane. The resulting filtrate serves as the test solution.

**1.2 Preparation of reference solution**

Accurately weigh appropriate amounts of caffeic acid, rosmarinic acid, schaftoside, and isoschaftoside reference standards. Dissolve each in a small amount of DMSO, then dilute with methanol to prepare stock solutions with a concentration of 1 mg·mL⁻¹. Accurately pipette aliquots of the stock solutions, mix them, and dilute to prepare mixed reference solutions with concentrations of 5, 25, 50, 100, and 200 μg·mL⁻¹.

**1.3 Selection of quantitative marker compounds**

The selection of caffeic acid, schaftoside, isoschaftoside, and rosmarinic acid as representative metabolites was based on both their documented biological relevance to liver protection and fibrosis, as well as their detectability in ILW. These compounds have been previously reported to be absorbed into the bloodstream following oral administration (Chen et al., 2024; Qin et al., 2024), supporting their pharmacological relevance. Caffeic acid has been shown to alleviate liver injury and oxidative stress via activation of the Nrf2/Keap1 signaling pathway and to reduce lipid accumulation in hepatocytes (Zhang et al., 2024; Reyes et al., 1995). Rosmarinic acid is known to inhibit hepatic stellate cell proliferation and suppress the expression of fibrosis-related markers, contributing to its anti-fibrotic potential (Elufioye & Habtemariam, 2019). Schaftoside has demonstrated efficacy in reducing oxidative stress, attenuating liver fibrosis, and regulating ferroptosis-related pathways (Yu et al., 2024). Isoschaftoside has been reported to suppress TNF-α release during M1 macrophage polarization and to alleviate symptoms of non-alcoholic fatty liver disease (NAFLD) (Abe, 2025). Taken together, these metabolites were taken as marker compounds in Quantitative analysis.

**1.4 Chromatographic conditions**

Chromatographic separation was carried out using an Agilent 1290 ultra-high-performance liquid chromatography (UPLC) system. Column: Waters ACQUITY UPLC HSS T3 column (2.1 mm × 100 mm, 1.8 μm); Mobile phase: 0.1% formic acid in water (A) and methanol (B), with gradient elution (0–42 min, 18.0%–49.5% B); Flow rate: 0.3 mL·min⁻¹; Column temperature: 30 ℃. Injection volume: 2 μL; Detection wavelength: 254 nm.

**1.5 Standard curve and Quantification results**

The chromatograms of ILW and mixed reference substances were shown in **Figure S1**. The standard curves of each reference substance are shown in **Table S4**. The contents of the index components in the ILW are as follows: caffeic acid, 1.10 mg·g⁻¹; schaftoside, 0.12 mg·g⁻¹; isoschaftoside, 0.18 mg·g⁻¹; and rosmarinic acid, 5.13 mg·g⁻¹.

**2 Fingerprint analysis of ILW samples**

**2.1 Preparation of ILW samples**

Twelve batches of *I. lophanthoides (Herba Isodonis Lophanthoidis)* (Batch Nos.: 20211011, 20211012, 20211013, 20211121, 20211122, 20211126, 20220313, 20220315, 20220316, 20220726, 20220728, 20220729) were sourced from Guangxi Xianzhu Traditional Chinese Medicine Technology Co., Ltd. (Nanning, China), Dashenlin Pharmaceutical Group Co., Ltd. (Yulin, China), Kangmei Pharmaceutical Co., Ltd. (Jieyang, China), and Qingping Traditional Chinese Medicine Market (Guangzhou, China)(**Table S5**).

Each batch was extracted following a standardized protocol. Briefly, 200 g of the dried botanical drug was decocted twice with 1600 mL of water, each for 1.5 hours. The decoctions were filtered through a 200-mesh gauze, the filtrates were combined and then concentrated under reduced pressure to yield the final extract.

**2.2 Chromatographic conditions**

Chromatographic analysis was performed using an Agilent 1290 Ultra-high-performance liquid chromatography (UPLC) system equipped with a Waters ACQUITY UPLC HSS T3 column (2.1 mm × 100 mm, 1.8 μm). The mobile phase consisted of 0.1% formic acid in water (solvent A) and methanol (solvent B), with a linear gradient elution (0–42 min, 18.0%–49.5% B). The flow rate was set at 0.3 mL/min, the column temperature was maintained at 30 ℃, and the injection volume was 2 μL. Detection was carried out at a wavelength of 254 nm.

**2.3 UPLC fingerprint**

Chromatographic data were processed using the Similarity Evaluation System for Chromatographic Fingerprint of Traditional Chinese Medicine (Version 2012.130723, National Pharmacopoeia Commission of China). Fingerprints were analyzed based on retention time alignment, peak matching, and similarity evaluation. A reference fingerprint was generated using the median method, and each sample was compared to the reference to calculate similarity indices (**Figure S5**). As shown in **Table S6**, the similarity values among the twelve batches were all above 0.95, indicating high consistency and good repeatability. These results suggest that the preparation process of ILW is stable, reasonable, and reproducible.

**3. Metabolomics analysis condition**

**3.1 Sample preparation**

Liver samples from mice in the control group, model group, and ILW-H group (n=8) were selected for metabolomics analysis. A total of 50 mg of liver tissue was weighed and placed in a 2 mL centrifuge tube. To this, 500 μL of precooled 70% methanol aqueous solution (at -20 ℃) was added, and the mixture was vortexed for 3 minutes. The samples were then centrifuged at 4 ℃ and 12,000 rpm for 10 minutes. After centrifugation, 300 μL of the supernatant was transferred to a 1.5 mL centrifuge tube and stored at -20 ℃ for 30 minutes. Following this, the samples were centrifuged again at 4 ℃ and 12,000 rpm for 10 minutes. Finally, 200 μL of the supernatant was collected and passed through a protein precipitation plate for further analysis. Quality control (QC) samples were prepared by combining 20 μl of each sample.

**3.2 Chromatographic and mass spectrometry conditions**

Chromatographic separations and data acquisition were performed using a SCIEX QTRAP 6500+ LC-MS/MS system. For the T3 method, chromatographic separation was performed using a Waters Acquity UPLC HSS T3 C18 column (100 mm × 2.1 mm, 1.8 µm). The mobile phases consisted of 0.05% formic acid in water (A) and 0.05% formic acid in acetonitrile (B). A gradient elution program was applied as follows: 0–8 min, 5%–95% B; 8–9.5 min, 95% B; 9.5–9.6 min, 95%–5% B; 9.6–12 min, 5% B. The total running time was 12 minutes. The injection volume was 2 µL, and the flow rate was set to 0.35 mL·min⁻¹. The column temperature was maintained at 40 ℃.

For the amide method, separation was achieved using an ACQUITY UPLC BEH Amide column (100 mm × 2.1 mm, 1.7 µm). The mobile phases were ultrapure water containing 10 mM ammonium acetate and 0.3% ammonia (A), and a 90% acetonitrile/water (V/V) mixture (B). The gradient elution program was as follows: 0–1.2 min, 5%–95% B; 1.2–8 min, 30%–70% B; 9.0–11 min, 70%–50% B; 11.1–15 min, 5%–95% B. The total running time was 15 minutes. The injection volume was 2 µL, and the flow rate was set to 0.4 mL·min⁻¹. The column temperature was maintained at 40 ℃.

Mass spectrometry was performed using electrospray ionization (ESI) in positive and negative ion modes. The ion source temperature was set to 550 ℃, with a capillary voltage of 5.5 kV for positive ion mode and 4.5 kV for negative ion mode. The curtain gas pressure was maintained at 35 psi. Data was collected in dynamic multiple reaction monitoring (MRM) mode. The ion pair information was obtained from AB SCIEX (USA).

**3.3 Data processing**

The raw data obtained from UPLC-MS/MS mass spectrometry were processed for peak extraction, alignment, and chromatographic peak integration using Analyst 1.6.3 and MultiQuant 3.03 software. Principal component analysis (PCA) was performed using the MetaboAnalyst 6.0 platform (https://www.metaboanalyst.ca). Differential metabolites were identified based on the criteria of *p*-Value < 0.05, fold change > 1.5, or fold change < 0.67. Significantly differential metabolites between the groups were visualized using a Venn diagram, generated by Venny 2.1 (https://bioinfogp.cnb.csic.es/tools/venny/). The common differential metabolites were subsequently imported into MetaboAnalyst 6.0 for KEGG metabolic pathway enrichment analysis and visualization. Besides, pearson correlation analysis was conducted on the QC samples.

**Reference**

Abe, T. (2025). Isoschaftoside in fig leaf tea alleviates nonalcoholic fatty liver disease in mice via the regulation of macrophage polarity. *Nutrients* 17(5), 757. doi:10.3390/nu17050757.

Chen, K. K., Dai, J., Guo, Z. H., Liang, J., Tan, Y., Zeng, Y. L., Luo, X., Guo, J. C. (2024). A qualitative detection method for the blood-absorbed and liver-absorbed components of *Isodon lophanthoides*. CN Patent No CN118604204A, China.

Chen, X., Liao, R. N., and Xie, Q. L. (2001). Abietane diterpenes from *Rabdosia serra* (maxim) hara. *J Chem Res.* 2001(4): 148-149. doi: 10.3184/030823401103169315.

Elufioye, T. O., and Habtemariam, S. (2019). Hepatoprotective effects of rosmarinic acid: insight into its mechanisms of action. *Biomed Pharmacother.* 112, 108600. doi:10.1016/j.biopha.2019.108600.

Guo, Z. H., Zheng, X. Y., Liang, J., Tan, Y., Qin, C. P., Guo, J. C., Li, Y. H., Wei, Z. Y., and Chen, K. K. (2022). Rapid identification of chemical constituents in Abrus cantoniensis Hance by UPLC-Q-TOF/MS combined with UNIFI platform. *China Pharm.* 33(23), 2852-2857. doi: 10.6039/j.issn.1001-0408.2022.23.07.

Jiang L, Belwal T, Huang H, Ge, Z. W., Limwachiranon, J., Zhao, Y. C., Li, L., Ren, G. P., and Luo, Z. S. (2019). Extraction and characterization of phenolic compounds from bamboo shoot shell under optimized ultrasonic-assisted conditions: A potential source of nutraceutical compounds. *Food Bioprocess Tech.* 2019, 12: 1741-1755. doi: 10.1007/s11947-019-02321-y.

Li, X, Xiao, W. L., Pu, J. X., Ban, L. L., Shen, Y. H., Weng, Z. Y., Li, S. H., and Sun, H. D. (2006). Cytotoxic ent-kaurene diterpenoids from *Isodon phyllostachys*. *Phytochemistry* 67(13): 1336-1340. doi: 10.1016/j.phytochem.2006.05.002.

Li, C., Wang, R. H., Wang, D. Z., Mao, J., Liu, S. D., and Zhang, J. X. (2017). Determination of coumarin and its metabolite in mouse blood with UPLC-orbitrap high resolution mass spectrometry. *J Henan Norm Univ Nat Sci Ed,* 2017, 45(2): 38-42. doi: 10.16366/j.cnki.1000-2367.2017.02.008.

Liu, Y. N., Hu, M. T., Qian J, Wang, Y., and Wang, S. F. (2021). Characterization of the chemical constituents of Jie-Geng-Tang and the metabolites in the serums and lungs of mice after oral administration by LC-Q-TOF-MS. *Chin J Nat Med.* 19(4): 284-294. doi: 10.1016/S1875-5364(21)60028-6.

Liu, Y., Ding, X. Y., Wang, N., Gao, Y., Yang, L. F., Lyu, J., Han, L. W., Fu, C. S., and Zhao, B. N. (2024). Component characterization of *Salvia miltiorrhiza* extracts based on UPLC-Q-Exactive Plus-Orbitrap MS and analysis of pharmacodynamic material basis related to spectral effect of its antithrombotic activity. *Chin Tradit Herb Drugs.* 55(5): 1609-1619. doi:10.7501/j.issn.0253-2670.2024.05.019.

Qin, P., Su, Y. J., Chen, Y. M., Zheng, R. Y., Zhong, J. N., Ye, X. Y., Ge, Y. W., and Chen, A. L. (2024). Chemical constituents in three origins of *Rabdosia serra* and their administration serum analysis in hepatic fibrosis mice based on UHPLC-Q-Exactive Orbitrap MS. *Cent South Pharm.* 22(2): 296-301. doi: 10.7539/j.issn.1672-2981.2024.02.004.

Reyes, M. T., Mourelle, M., Hong, E., and Muriel, P. (1995). Caffeic acid prevents liver damage and ameliorates liver fibrosis induced by CCl4 in the rat. *Drug Dev Res.* 36(3), 125-128. doi: 10.1002/ddr.430360305.

Yang, Y., Zhao, H., Zhu, F. R., Liu, X. Y., Liu, Y., Zeng, F., and Liu, B. (2022). Analysis of isoflavones in Pueraria by UHPLC-Q-orbitrap HRMS and study on α-glucosidase inhibitory activity. *Foods* 11(21): 3523. doi: 10.3390/foods11213523.

Yu, Y., Liang, J. W., Yuan, Z. X., Wang, A. P., Liu, X. X., Chen, Y., Zhang, M., Gao, Y. N., Zhang, H. Y., and Liu, Y. (2024). Bioactive compound schaftoside from Clinacanthus nutans attenuates acute liver injury by inhibiting ferroptosis through activation the Nrf2/GPX4 pathway. *J Ethnopharmacol.* 328, 118135. doi:10.1016/j.jep.2024.118135.

Zhang, J., Ouyang, H., Gu, X., Dong, S., Lu, B., Huang, Z., and Ji, L. (2024). Caffeic acid ameliorates metabolic dysfunction-associated steatotic liver disease via alleviating oxidative damage and lipid accumulation in hepatocytes through activating Nrf2 via targeting Keap1. *Free Radic. Biol. Med.* 224, 352-365. doi: 10.1016/j.freeradbiomed.2024.08.038.

**Figure S1** Correlation analysis of metabolomics QC samples

**Figure S2** MS/MS spectra and proposed fragmentation pathway of vicenin-2

**Figure S3** Chromatograms of ILW and official reference standard of the powdered plant material (ORSPP). ILW at 254 nm (A); ORSPP at 254 nm (B); ILW at 270 nm (C); ORSPP at 270 nm (D); ILW at 300 nm (E); ORSPP at 300 nm (F)

**Figure S4** Chromatograms of ILW (A) and a mixture of reference standards (B)

**Figure S5** UPLC fingerprint analysis of ILW

**Figure S6** Heat map analysis of DEGs

**Figure S7** Heat map analysis of DEPs

**Figure S8** Concentrations of inosine in the Control, Model, and ILW-H groups (n = 8). Data presented as mean ± SEM.

**Figure S9** Relative protein abundance of Nt5e (n = 3). Data presented as mean ± SEM; ^*^*p* < 0.05, compared to Control group; ^#^*p* < 0.05, compared to Model group

**Table S1** Detailed information of the crude drug

**Table S2** Detailed mass spectrometry parameters for seven validated metabolites

**Table S3** The linear equation and LOQ of seven validated metabolites

**Table S4** Identification of ILW metabolites by UPLC-Q-TOF/MS

**Table S5** The linear equation of caffeic acid, schaftoside, isoschaftoside and rosmarinic acid

**Table S6** Batch information of twelve *I. lophanthoides* water extracts

**Table S7** Similarity analysis results between ILW samples and the reference fingerprint

**Table S8** The detailed information of shared DEGs between Model vs. Control and ILW-H vs. Model comparisons

**Table S9** The detailed information of shared DEPs between Model *vs.* Control and ILW-H *vs.* Model comparisons


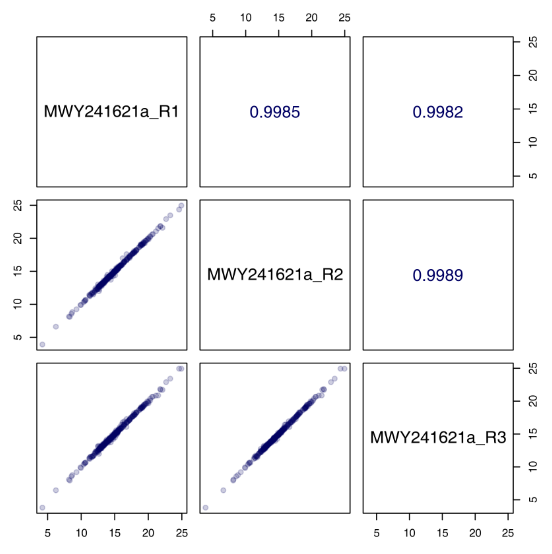


Figure S1 Correlation analysis of metabolomics QC samples


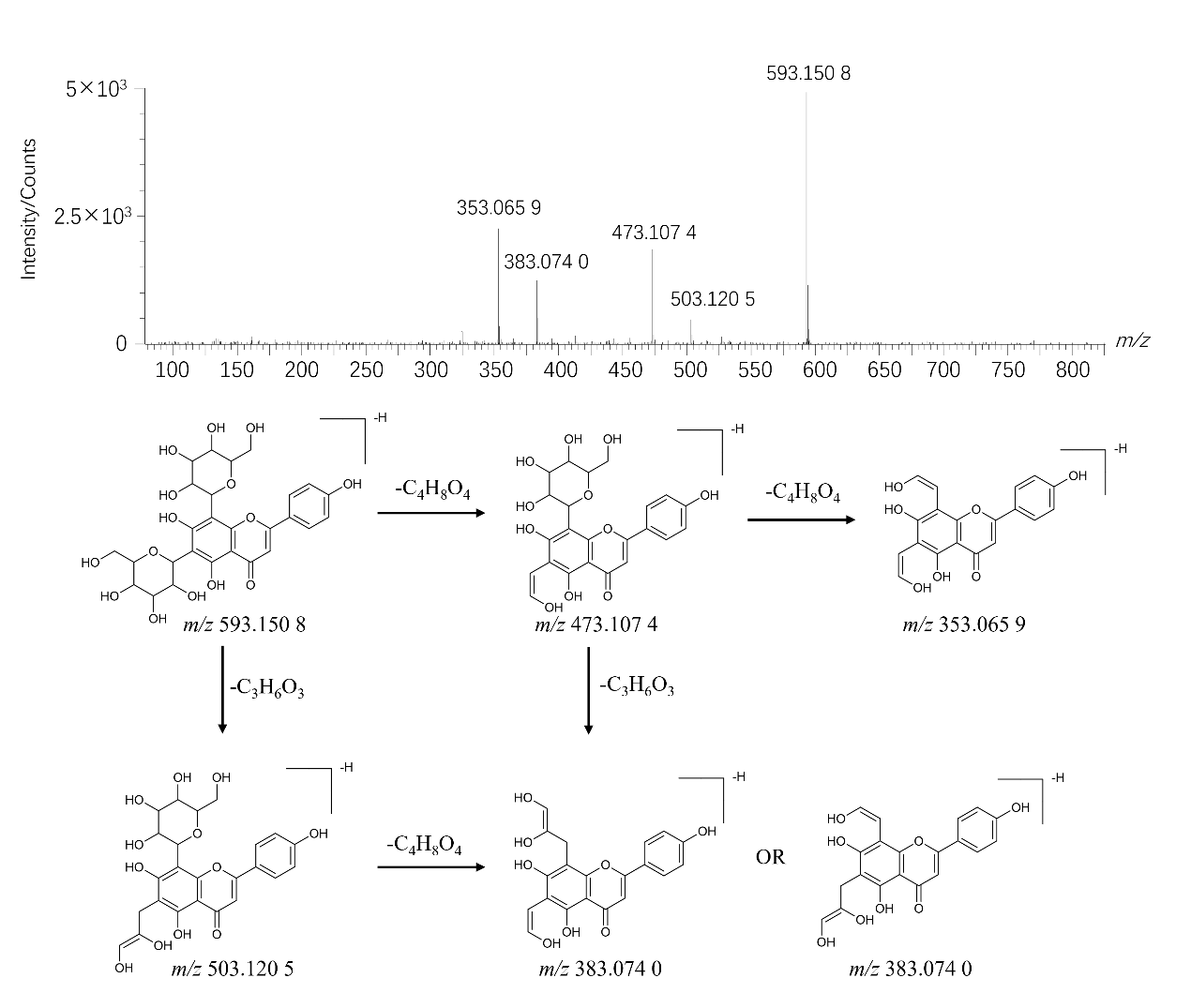


Figure S2 MS/MS spectra and proposed fragmentation pathway of vicenin-2


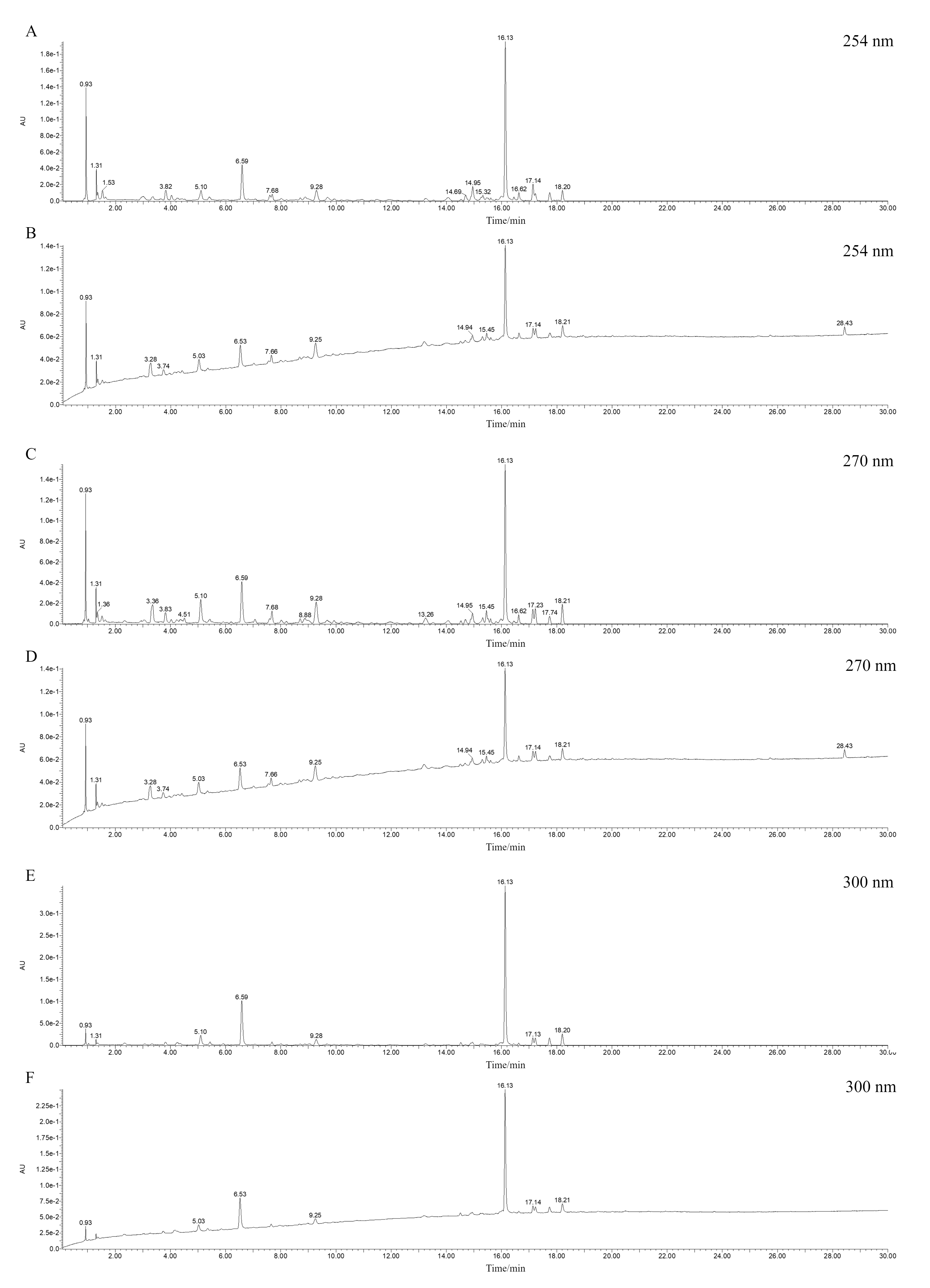


Figure S3 Chromatograms of ILW and official reference standard of the powdered plant material (ORSPP). ILW at 254 nm (A); ORSPP at 254 nm (B); ILW at 270 nm (C); ORSPP at 270 nm (D); ILW at 300 nm (E); ORSPP at 300 nm (F).


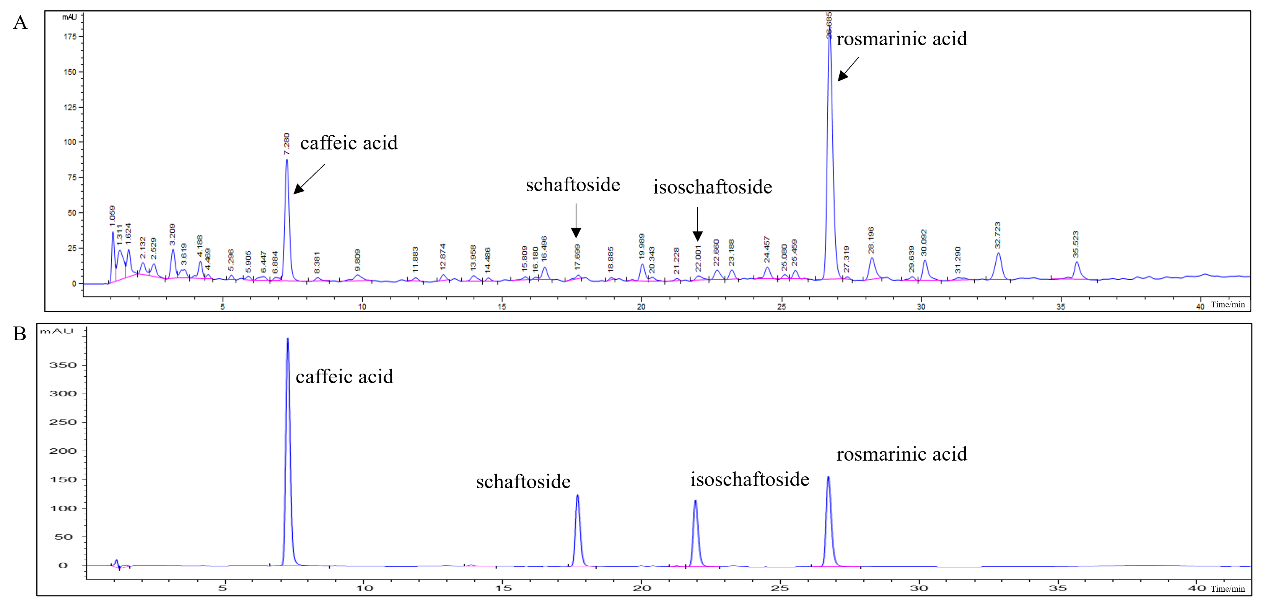


Figure S4 Chromatograms of ILW (A) and a mixture of reference standards (B)


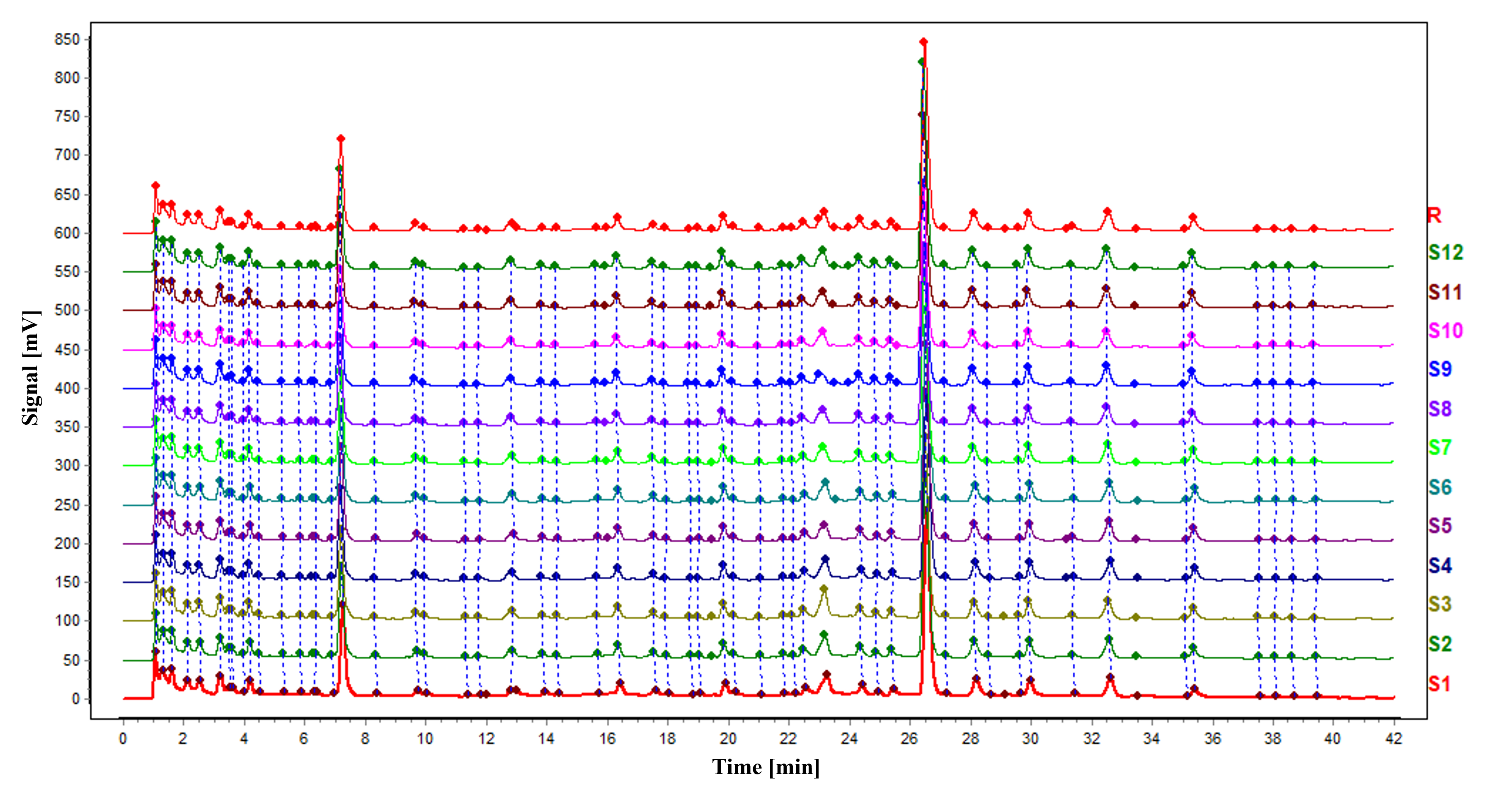


Figure S5 UPLC fingerprint analysis of ILW


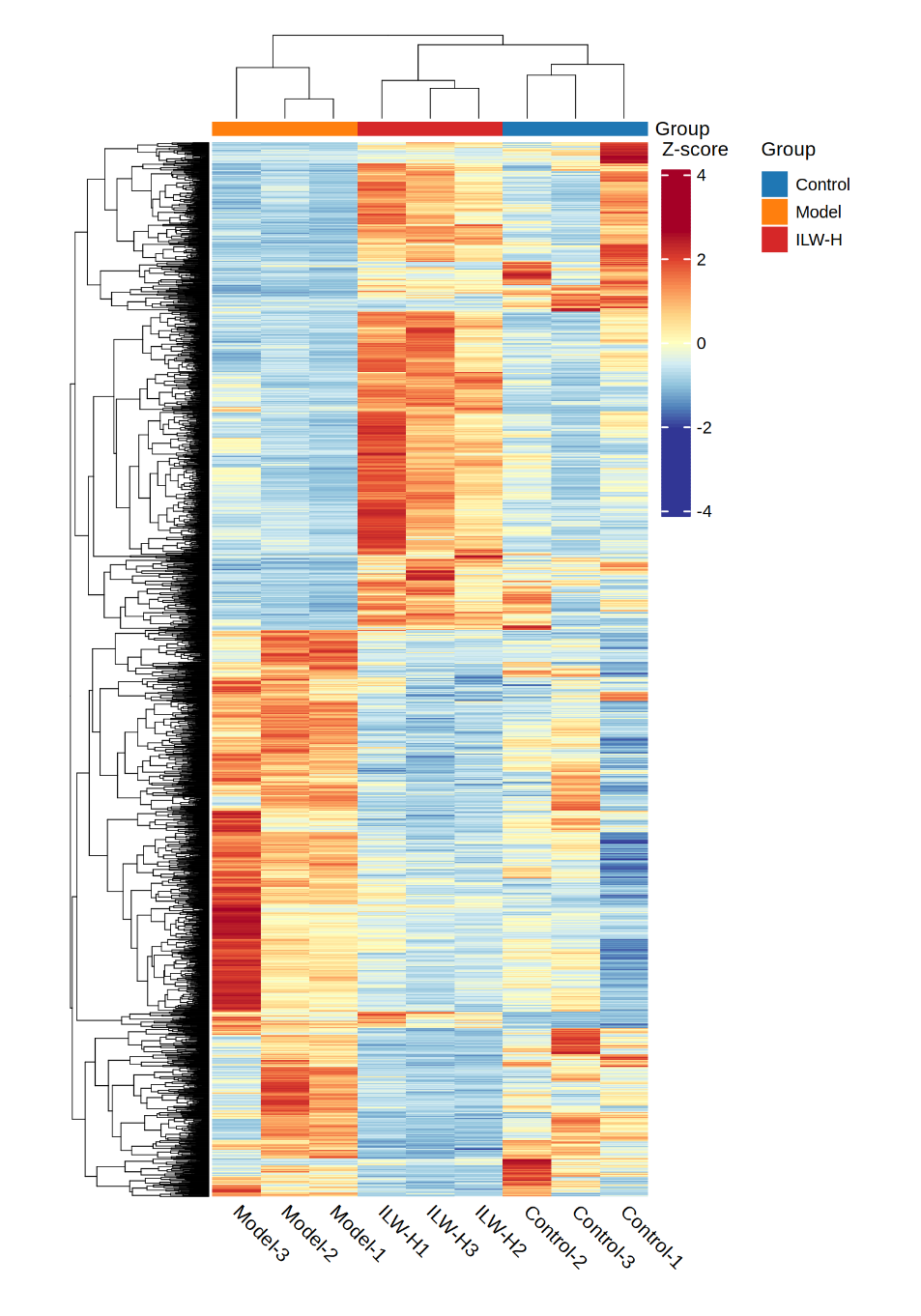


Figure S6 Heat map analysis of DEGs


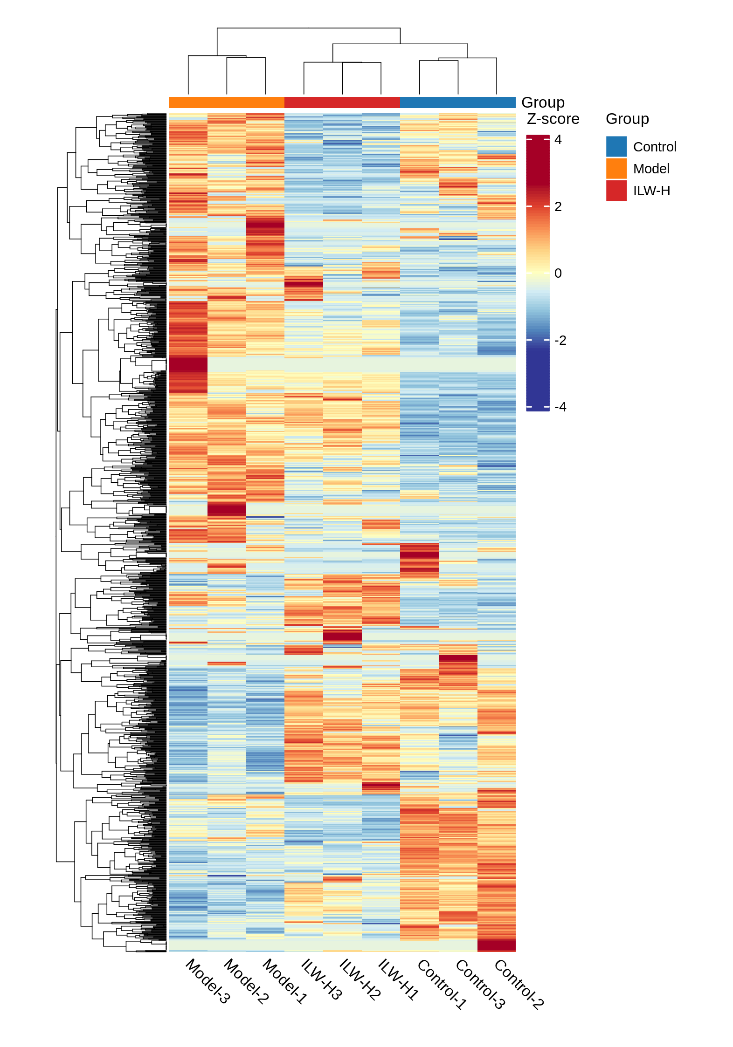


Figure S7 Heat map analysis of DEP


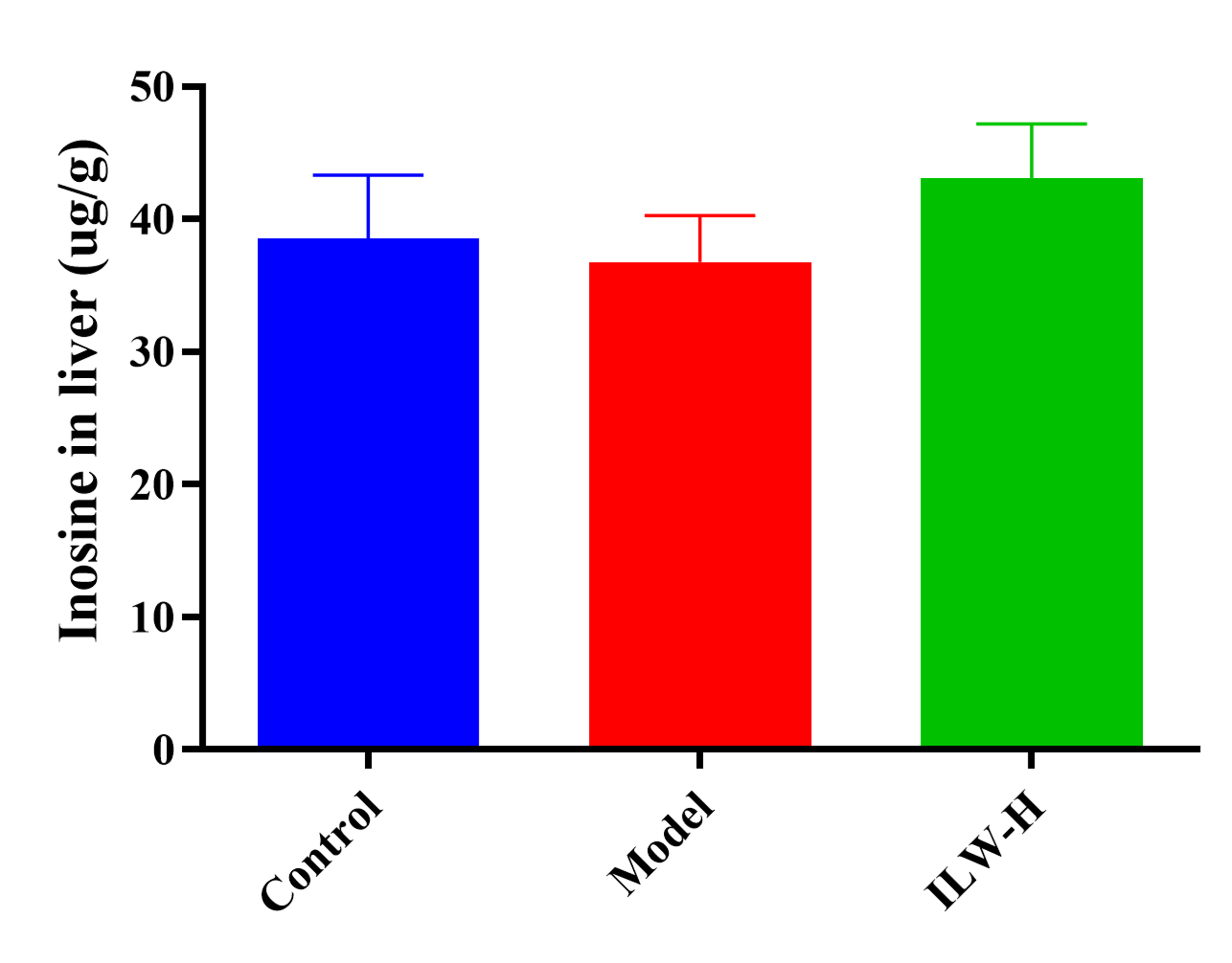


Figure S8 Concentrations of inosine in the Control, Model, and ILW-H groups (n = 8). Data presented as mean ± SEM.


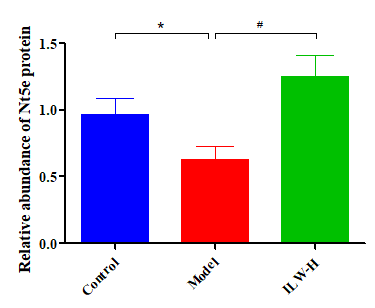


Figure S9 Relative protein abundance of Nt5e (n = 3). Data presented as mean ± SEM; ^*^*p* < 0.05, compared to Control group; ^#^*p* < 0.05, compared to Model group

Table S1 Detailed information of the crude drug

| Voucher number | Name | Authentication | Origin | Voucher specimen |
| --- | --- | --- | --- | --- |
| No. GUCM2021013 | Herba Isodonis Lophanthoidis | Dried aerial parts (stems and leaves) of *Isodon lophanthoides* (Buch.-Ham. Ex D. Don) H. Hara | Puning City, Guangdong, China | 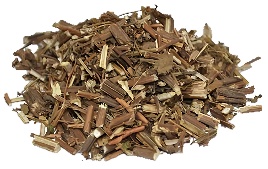 |

Table S2 Detailed mass spectrometry parameters for seven validated metabolites

| Compound | RT/min | Ionization | Precursor ion | Product ion | DP/eV | CE/eV |
| --- | --- | --- | --- | --- | --- | --- |
| N^6^-methyladenosine | 1.22 | [M+H]^+^ | 282.1 | 150.1 | 30 | 30 |
| Hypoxanthine | 1.23 | [M+H]^+^ | 137.04 | 110.2 | 60 | 20 |
| Adenine | 1.37 | [M-H]^−^ | 134.0 | 107.0 | 50 | 24 |
| Adenosine | 1.43 | [M+H]^+^ | 268.1 | 136.1 | 33 | 22 |
| Inosine | 1.45 | [M-H]^−^ | 267.0 | 135.0 | 20 | 30 |
| AMP | 5.38 | [M-H]^−^ | 345.9 | 79.0 | 60 | 24 |
| IMP | 5.76 | [M-H]^−^ | 346.9 | 78.9 | 60 | 24 |

DP: declustering potential; CE: collision energy.

Table S3 The linear equation and LOQ of seven validated metabolites

| Compound | Linear equation | r | Linear range(ng/mL) | LOQ(ng/mL) |
| --- | --- | --- | --- | --- |
| N^6^-methyladenosine | *y* = 7.9468e5 *x* + 1.2527e6 | 0.9912 | 1~100 | 1 |
| Hypoxanthine | *y* = 1708.6509 *x* + 29619.3591 | 0.9922 | 20~500 | 20 |
| Adenine | *y* = 2.2996e5 *x* + 3.4941e5 | 0.9931 | 0.2~20000 | 0.2 |
| Adenosine | *y* = 1.1112e5 *x* + 18384.4199 | 0.9943 | 2~200 | 2 |
| Inosine | *y* = 4.5279e4 *x* + 8255.0922 | 0.9994 | 5~200 | 5 |
| AMP | *y* = 6.3216e4 *x* – 3.0570e6 | 0.9956 | 2~20000 | 2 |
| IMP | *y* = 16181.0950 *x* – 1.8269e6 | 0.9963 | 100~5000 | 100 |

Table S4 Identification of ILW metabolites by UPLC-Q-TOF/MS

| **No.** | **RT/min** | **Formula** | **Measured** | **ppm** | **MS/MS** | **Name** | **Reference** |
| --- | --- | --- | --- | --- | --- | --- | --- |
| **O1** | 0.99 | C_4_H_6_O_5_ | 133.013 4 [M-H]^−^ | -2.1 | 115.003 7 [M-H-H_2_O]^−^ | Malic acid | Pubchem database |
| **O2** | 1.36 | C_6_H_8_O_7_ | 191.019 3 [M-H]^−^ | -0.5 | 128.034 1 [M-H-CO_2_-H_2_O]^−^, 111.007 2 [M-H-CO_2_-2H_2_O]^−^ | Citric acid | (Guo et al., 2022) |
| **O3** | 3.41 | C_9_H_10_O_5_ | 197.045 5 [M-H]^−^ | 2.5 | 179.034 2 [M-H- H_2_O]^−^, 135.045 1 [M-H-H_2_O-CO_2_]^−^ | Danshensu | (Liu et al., 2024) |
| **O4** | 3.70 | C_8_H_8_O_4_ | 167.033 9 [M-H]^−^ | 1 | 123.044 1 [M-H-CO_2_]^−^, 108.015 1 [M-H-CO_2_-CH_3_]^−^ | Vanillic acid | (Qin et al., 2024) |
| **O5** | 3.88 | C_7_H_6_O_4_ | 153.018 8 [M-H]^−^ | -0.1 | 109.027 7 [M-H-CO_2_]^−^ | Protocatechuic acid | Pubchem database |
| **O6** | 4.56 | C_9_H_10_O_4_ | 181.050 4 [M-H]^−^ | 1.7 | 163.039 1 [M-H-H_2_O]^−^、135.044 3 [M-H-H_2_O-CO]^−^、119.049 7 [M-H-H_2_O-CO_2_]^−^ | 4-Hydroxyphenyl lactic acid | MassBank Database |
| **O7** | 5.49 | C_15_H_18_O_9_ | 341.086 5 [M-H]^−^ | -2.3 | 179.034 2 [M-H-Glu]^−^、135.038 3 [M-H-Glu-CO_2_]^−^ | Caffeic acid-3-O-β-D-glucoside | HMDB Database |
| **O8** | 5.92 | C_12_H_14_O_8_ | 285.060 1 [M-H]^−^ | 0.7 | 153.016 1 [M-H-C_5_H_8_O_4_]^−^, 109.030 1 [M-H-C_5_H_8_O_4_-CO_2_]^−^ | Uralenneoside | (Liu et al., 2021) |
| **O9** | 6.63 | C_9_H_8_O_4_ | 179.034 3 [M-H]^−^ | -0.6 | 135.044 5 [M-H-CO_2_]^−^, 107.048 3 [M-H-CO_2_-CO]^−^ | Caffeic acid | (Qin et al., 2024) |
| **O10** | 14.57 | C_24_H_26_O_13_ | 521.129 6[M-H]^−^ | 0.2 | 359.073 9 [M-H-Glu]^−^, 323.078 3 [M-H-Glu-2H_2_O]^−^, 197.044 6 [M-H-Glu-C_9_H_7_O_3_]^−^, 179.035 9 [M-H-Glu-C_9_H_8_O_4_]^−^ | Salviaflaside | Pubchem database |
| **O11** | 15.01 | C_36_H_30_O_16_ | 717.149 0 [M-H]^−^ | 4.7 | 519.102 8 [M-H-C_9_H_10_O_5_]^−^, 321.035 4 [M-H-2C_9_H_10_O_5_]^−^ | Salvianolic acid B isomer | (Liu et al., 2024) |
| **O12** | 16.21 | C_18_H_16_O_8_ | 359.077 9 [M-H]^−^ | 3.3 | 197.045 6 [M-H-C_9_H_7_O_3_]^−^, 179.034 2 [M-H-C_9_H_8_O_4_]^−^, 161.024 1 [M-H-C_9_H_8_O_4_-H_2_O]^−^ | Rosmarinic acid | Reference substance |
| **O13** | 16.50 | C_26_H_22_O_10_ | 493.112 5 [M-H]^−^ | -2.0 | 295.0610 0 [M-H-C_9_H_10_O_5_]^−^, 267.062 8 [M-H-C_9_H_10_O_5_-H_2_O]^−^ | Salvianolic acid A | (Liu et al., 2024) |
| **O14** | 17.20 | C_36_H_30_O_16_ | 717.148 6 [M-H]^−^ | 4.2 | 519.095 1 [M-H-C_9_H_10_O_5_]^−^, 321.040 2 [M-H-2C_9_H_10_O_5_]- | Salvianolic acid B | (Liu et al., 2024) |

F represents flavonoids; O represents organic acids; T represents terpenes; Ot represents other categories.

Table S4 Identification of ILW metabolites by UPLC-Q-TOF/MS (*Cont.)*

| **No.** | **RT/min** | **Formula** | **Measured** | **ppm** | **MS/MS** | **Name** | **Reference** |
| --- | --- | --- | --- | --- | --- | --- | --- |
| **F1** | 7.75 | C_27_H_30_O_15_ | 593.150 8 [M-H]^−^ | 0.3 | 503.120 5 [M-H-C_3_H_6_O_3_]^−^, 473.107 4 [M-H-C_4_H_8_O_4_]^−^, 383.074 0 [M-H-C_4_H_8_O_4_-C_3_H_6_O_3_]^−^, 353.065 9 [M-H-2C_4_H_8_O_4_]^−^, | Vicenin-2 | Reference substance |
| **F2** | 9.34 | C_26_H_28_O_14_ | 563.139 9 [M-H]^−^ | -0.4 | 473.106 5 [M-H-C_3_H_6_O_3_]^−^, 443.096 9 [M-H-C_4_H_8_O_4_]^−^, 383.076 6 [M-H-C_4_H_8_O_4_-C_2_H_4_O_2_]^−^, 353.064 9 [M-H-C_4_H_8_O_4_-C_3_H_6_O_3_]^−^ | Schaftoside | Reference substance |
| **F3** | 10.91 | C_26_H_28_O_14_ | 563.140 1 [M-H]^−^ | -0.2 | 473.103 2 [M-H-C_3_H_6_O_3_]^−^, 443.097 2 [M-H-C_4_H_8_O_4_]^−^, 383.081 8 [M-H-C_4_H_8_O_4_-C_2_H_4_O_2_]^−^, 353.061 0 [M-H-C_4_H_8_O_4_-C_3_H_6_O_3_]^−^ | Isoschaftoside | Reference substance |
| **F4** | 11.93 | C_27_H_30_O_16_ | 609.148 3 [M-H] ^−^ | 4.4 | 301.021 4 [M-H-Glu-Rha]^−^, 151.0397[C_7_H_4_O_4_]^−^ | Rutin | (Qin et al., 2024) |
| **F5** | 12.03 | C_21_H_20_O_10_ | 433.113 3 [M+H]^+^ | -0.1 | 415.104 9 [M+H-H_2_O]^+^, 313.109 2 [M+H-C_4_H_8_O_4_]^+^, 295.063 9 [M+H-C_4_H_8_O_4_-H_2_O]^+^ | Vitexin | Reference substance |
| **F6** | 12.77 | C_21_H_20_O_10_ | 433.113 6 [M+H]^+^ | 0.2 | 313.061 6 [M+H-C_4_H_8_O_4_]^+^, 295.059 6 [M+H-C_4_H_8_O_4_-H_2_O]^+^ | Isovitexin | Reference substance |
| **F7** | 13.30 | C_25_H_26_O_13_ | 533.133 6 [M-H]^−^ | 3.4 | 473.110 4 [M-H-C_2_H_4_O_2_]^−^, 443.104 2 [M-H-C_3_H_6_O_3_]^−^, 383.079 8 [M-H-C_3_H_6_O_3_-C_2_H_4_O_2_]^−^, 353.65 1 [M-H-C_3_H_6_O_3_-C_3_H_6_O_3_]^−^ | Apigenin-6,8-di-C-α-L-arabinoside | (Jiang et al., 2019) |
| **F8** | 17.30 | C_26_H_28_O_13_ | 549.160 2 [M+H]^+^ | 3.5 | 521.106 6 [M-H-CO]^+^, 417.250 5 [M-H-Xyl]^+^, 399.396 3 [M-H-Xyl-H_2_O]^+^ | Puerarin-6″-O-xyloside | (Yang et al., 2022) |
| **F9** | 17.80 | C_18_H_16_O_7_ | 343.080 9 [M-H]^−^ | 1.3 | 329.15108 [M-H-CH_3_]^−^, 313.120 7 [M-H-2CH_3_]^−^, 299.061 1 [M-H-2CH_3_-CH_2_]^−^ | Eupatilin | Pubchem database |
| **T1** | 15.52 | C_26_H_32_O_12_ | 535.181 5 [M-H]^−^ | -0.2 | 355.118 2 [M-H-Glu-H_2_O]^−^, 337.090 2 [M-H-Glu-2H_2_O]^−^ | Fibrauretinoside A | Pubchem database |
| **T2** | 22.35 | C_20_H_24_O_5_ | 343.153 7 [M-H]^−^ | -2.3 | 328.126 8 [M-H-CH_3_]^−^, 313.091 8 [M-H-2CH_3_]^−^, 295.101 5 [M-H-2CH_3_-H_2_O]^−^ | Laxiflorin B | (Qin et al., 2024) |
| **T3** | 24.89 | C_22_H_28_O_6_ | 387.180 2 [M-H]^−^ | -1.5 | 345.159 2[M-H-CH_2_CO]^−^, 327.160 8[M-H-CH_2_CO-H_2_O]^−^, 309.191 9 [M-H-CH_2_CO-2H_2_O]^−^ | Maoecrystal A | (Qin et al., 2024) |

F represents flavonoids; O represents organic acids; T represents terpenes; Ot represents other categories.

Table S4 Identification of ILW metabolites by UPLC-Q-TOF/MS (*Cont.)*

| **No.** | **RT/min** | **Formula** | **Measured** | **ppm** | **MS/MS** | **Name** | **Reference** |
| --- | --- | --- | --- | --- | --- | --- | --- |
| **T4** | 25.98 | C_20_H_26_O_4_ | 329.174 7 [M-H]^−^ | -2.4 | 314.150 5 [M-H-CH_3_]^−^, 299.127 5 [M-H-2CH_3_]^−^ | 6,12,15-trihydroxy-5,8,11,13-abietetriene-7-one | (Chen et al., 2001) |
| **T5** | 26.03 | C_30_H_46_O_6_ | 501.321 9 [M-H]^−^ | 0.6 | 455.317 2 [M-H-H_2_O-CO]^−^, 439.317 8 [M-H-CO_2_-H_2_O]^−^ | Medicagenic acid | MassBank database |
| **T6** | 26.97 | C_20_H_28_O_5_ | 347.186 7 [M-H]^−^ | 2.6 | 329.249 9 [M-H-H_2_O]^−^ | Amethystoidin A | (Li et al., 2006) |
| **Ot1** | 1.47 | C_7_H_11_NO_5_ | 188.057 1 [M-H]^−^ | 4.8 | 144.066 5 [M-H-CO_2_]^−^, 128.035 9 [M-H-H_2_O-CH_2_CO]^−^, 102.055 9 [M-H-CH_2_CO-CO_2_]^−^ | N-Acetyl-L-glutamic acid | Pubchem database |
| **Ot2** | 5.16 | C_7_H_6_O_3_ | 137.023 7 [M-H]^−^ | -2.8 | 109.028 2 [M-H-CO]^−^ | Protocatechualdehyde | (Qin et al., 2024) |
| **Ot3** | 6.59 | C_9_H_6_O_3_ | 163.038 7 [M+H]^+^ | -4.9 | 145.027 3 [M+H-H_2_O]^+^, 135.044 2 [M+H-CO]^+^, 119.052 0 [M+H-CO_2_]^+^ | 7-Hydroxycoumarin | (Li et al., 2017) |

F represents flavonoids; O represents organic acids; T represents terpenes; Ot represents other categories.

Table S5 Linear relationship of each reference substance

| Compounds | Regression equation | r | Linear range /μg·mL^-1^ |
| --- | --- | --- | --- |
| Caffeic acid | *Y* = 22.196 9 *X－*33.400 5 | 0.999 6 | 5.00～200 |
| Rosmarinic acid | *Y* = 10.511 2 *X－*58.072 6 | 0.997 3 | 5.00～200 |
| Schaftoside | *Y* = 7.584 8 *X－*13.271 0 | 0.999 5 | 5.00～200 |
| Isoschaftoside | *Y* = 7.280 9 *X－*12.631 4 | 0.999 6 | 5.00～200 |

Table S6 Batch information of twelve *I. lophanthoides* water extracts

| No. | ILW Batchs | *I. Lophanthoidis*  Batchs | Origin | No. | ILW Batchs | *I. Lophanthoidis*  Batchs | Origin |
| --- | --- | --- | --- | --- | --- | --- | --- |
| S1 | No.202209013 | No. 20211011 | Puning City, Guangdong, China | S7 | No.202210006 | No. 20220313 | Guagnzhou City, Guangdong, China |
| S2 | No.202209015 | No. 20211012 | Puning City, Guangdong, China | S8 | No.202210011 | No. 20220315 | Guagnzhou City, Guangdong, China |
| S3 | No.202209019 | No. 20211013 | Puning City, Guangdong, China | S9 | No.202210013 | No. 20220316 | Guagnzhou City, Guangdong, China |
| S4 | No.202209021 | No. 20211121 | Yulin City, Guagnxi, China | S10 | No.202210017 | No. 20220726 | Huizhou City, Guangdong, China |
| S5 | No.202209023 | No. 20211122 | Yulin City, Guagnxi, China | S11 | No.202210021 | No. 20220728 | Huizhou City, Guangdong, China |
| S6 | No.202209027 | No. 20211126 | Yulin City, Guagnxi, China | S12 | No.202210023 | No. 20220729 | Huizhou City, Guangdong, China |

Table S7 Similarity analysis results between ILW samples and the reference fingerprint

| No. | Reference fingerprint | No. | Reference fingerprint |
| --- | --- | --- | --- |
| S1 | 0.958 | S7 | 0.981 |
| S2 | 0.959 | S8 | 0.982 |
| S3 | 0.958 | S9 | 0.959 |
| S4 | 0.981 | S10 | 0.971 |
| S5 | 0.959 | S11 | 0.972 |
| S6 | 0.981 | S12 | 0.959 |

Table S8 The detailed information on shared DEGs

| Gene IDs | Model vs. Control | | | ILW-H vs. Model | | |
| --- | --- | --- | --- | --- | --- | --- |
|  | log_2_FoldChange | FDR | Trend | log_2_FoldChange | FDR | Trend |
| Cdh1 | 2.0910 | 0.0019 | up | -2.3971 | 0.0000 | down |
| Sult5a1 | -3.4096 | 0.0003 | down | 3.7037 | 0.0000 | up |
| Cfp | -2.1748 | 0.0024 | down | 0.8808 | 0.0158 | up |
| Lrp3 | -1.7958 | 0.0010 | down | 1.2637 | 0.0001 | up |
| Rhoc | 1.6608 | 0.0079 | up | -1.3429 | 0.0000 | down |
| Cyp2c29 | -2.9148 | 0.0009 | down | 2.5830 | 0.0001 | up |
| Car11 | -5.4060 | 0.0113 | down | 3.4614 | 0.0031 | up |
| Ccl24 | -3.6477 | 0.0000 | down | 1.4657 | 0.0324 | up |
| Prlr | -2.3201 | 0.0003 | down | 1.5508 | 0.0001 | up |
| Cd163 | -4.0406 | 0.0002 | down | 2.4978 | 0.0003 | up |
| Spire2 | 4.6223 | 0.0001 | up | -3.6061 | 0.0006 | down |
| Clec4f | -3.9745 | 0.0000 | down | 2.6773 | 0.0000 | up |
| C8g | -1.2018 | 0.0174 | down | 1.0113 | 0.0204 | up |
| Ptgds | -3.4975 | 0.0000 | down | 4.0215 | 0.0000 | up |
| Arnt2 | 3.8146 | 0.0246 | up | -2.0501 | 0.0420 | down |
| Prune1 | 0.9802 | 0.0415 | up | -0.7786 | 0.0281 | down |
| Col20a1 | -1.7440 | 0.0383 | down | 1.7732 | 0.0002 | up |
| Tbx3 | -1.6538 | 0.0009 | down | 1.2617 | 0.0004 | up |
| Rab36 | 1.4267 | 0.0038 | up | -1.7501 | 0.0000 | down |
| Igfbp1 | 1.4248 | 0.0178 | up | -2.5405 | 0.0000 | down |
| Ncoa1 | 3.1247 | 0.0031 | up | -3.2915 | 0.0002 | down |
| Rasl10b | 3.8238 | 0.0079 | up | -3.4843 | 0.0000 | down |
| Cyp46a1 | -4.8620 | 0.0000 | down | 4.8923 | 0.0000 | up |
| Dct | -2.4360 | 0.0189 | down | 3.6555 | 0.0000 | up |
| Adcy4 | -2.9222 | 0.0001 | down | 1.7265 | 0.0000 | up |
| A1bg | -8.3178 | 0.0196 | down | 6.5358 | 0.0052 | up |
| Enpp2 | 1.7343 | 0.0019 | up | -2.2941 | 0.0000 | down |
| Btg3 | 2.7887 | 0.0031 | up | -1.7660 | 0.0198 | down |
| Ets2 | 1.1721 | 0.0031 | up | -0.8858 | 0.0000 | down |
| Slc15a2 | -7.2344 | 0.0319 | down | 7.9334 | 0.0000 | up |
| Ifngr2 | 1.0680 | 0.0242 | up | -1.4177 | 0.0000 | down |
| Scn8a | 2.6933 | 0.0016 | up | -2.8176 | 0.0000 | down |
| Cdkn1a | 2.9627 | 0.0003 | up | -2.3585 | 0.0000 | down |
| Rgn | -1.9209 | 0.0220 | down | 2.1503 | 0.0004 | up |
| Slc29a1 | -1.5116 | 0.0054 | down | 1.2562 | 0.0000 | up |
| Cyp39a1 | 1.7067 | 0.0148 | up | -1.0709 | 0.0118 | down |
| Epb41l3 | -2.8757 | 0.0035 | down | 1.8211 | 0.0068 | up |
| Cyp4f14 | -1.5147 | 0.0007 | down | 0.8789 | 0.0428 | up |
| Btnl2 | 3.6505 | 0.0310 | up | -3.5742 | 0.0130 | down |
| Spink1 | 5.0725 | 0.0000 | up | -4.2007 | 0.0000 | down |
| Csf1r | -1.9064 | 0.0130 | down | 0.8377 | 0.0284 | up |
| Slc25a45 | -1.5993 | 0.0000 | down | 1.1314 | 0.0001 | up |
| Cyp2c39 | -2.6600 | 0.0001 | down | 2.3866 | 0.0000 | up |
| Cyp2c40 | -4.9530 | 0.0000 | down | 1.4612 | 0.0175 | up |
| Dgka | -1.4276 | 0.0448 | down | 1.2715 | 0.0049 | up |
| Cyp2e1 | -2.3856 | 0.0000 | down | 1.8790 | 0.0003 | up |
| Cyp27a1 | -1.2350 | 0.0189 | down | 0.9134 | 0.0214 | up |
| Igfbp5 | -3.3565 | 0.0004 | down | 2.5072 | 0.0011 | up |
| Marco | -2.4812 | 0.0000 | down | -1.2358 | 0.0010 | down |
| Tagln2 | 1.6941 | 0.0016 | up | -1.5337 | 0.0000 | down |
| Plxna2 | 1.1751 | 0.0285 | up | -0.8513 | 0.0073 | down |
| Upp2 | -1.6914 | 0.0047 | down | 1.5854 | 0.0000 | up |
| Car1 | -3.1104 | 0.0281 | down | 4.3670 | 0.0000 | up |
| Cyp7a1 | -2.2717 | 0.0001 | down | 4.1827 | 0.0000 | up |
| Zfp37 | 3.7060 | 0.0012 | up | -3.2114 | 0.0062 | down |
| Enho | -1.1506 | 0.0034 | down | -1.3835 | 0.0286 | down |
| Gba2 | -1.1930 | 0.0107 | down | 1.2628 | 0.0001 | up |
| Npr2 | -3.9788 | 0.0000 | down | 2.8382 | 0.0000 | up |
| Mfsd2a | -1.6565 | 0.0000 | down | 2.3781 | 0.0000 | up |
| Tmem54 | 4.5993 | 0.0165 | up | -2.4579 | 0.0352 | down |
| Smpdl3b | 2.4716 | 0.0007 | up | -1.8617 | 0.0148 | down |
| Arhgef16 | 2.2855 | 0.0000 | up | -1.4596 | 0.0036 | down |
| Htra3 | 1.7368 | 0.0301 | up | 1.3830 | 0.0013 | up |
| Paics | 1.4406 | 0.0079 | up | -1.4967 | 0.0004 | down |
| Igf2bp3 | 2.7916 | 0.0002 | up | -1.7609 | 0.0041 | down |
| Nat8 | -2.4458 | 0.0405 | down | 3.7657 | 0.0000 | up |
| Slco1a4 | -1.3129 | 0.0380 | down | 2.1932 | 0.0000 | up |
| Ttll3 | -2.0451 | 0.0280 | down | 1.8450 | 0.0000 | up |
| Cand2 | 2.6378 | 0.0074 | up | -1.9435 | 0.0086 | down |
| Sult2a8 | -3.3809 | 0.0000 | down | 3.9422 | 0.0000 | up |
| Dmpk | -1.6288 | 0.0281 | down | 1.1233 | 0.0091 | up |
| Mfge8 | 1.9363 | 0.0174 | up | -0.8840 | 0.0438 | down |
| Syt3 | -3.6439 | 0.0028 | down | 3.3724 | 0.0000 | up |
| Slco2b1 | -1.3227 | 0.0159 | down | 0.8934 | 0.0103 | up |
| Denn2b | 1.2223 | 0.0166 | up | -1.4700 | 0.0000 | down |
| Porcn | -1.5249 | 0.0170 | down | 1.2855 | 0.0102 | up |
| Chic1 | -2.8111 | 0.0027 | down | 2.2442 | 0.0020 | up |
| Psmd10 | 1.0500 | 0.0303 | up | -1.3723 | 0.0001 | down |
| Adrb3 | -2.8569 | 0.0001 | down | 2.2697 | 0.0000 | up |
| Adgrg1 | 1.7584 | 0.0161 | up | -1.3663 | 0.0002 | down |
| Nxpe2 | -2.9226 | 0.0000 | down | 1.1586 | 0.0300 | up |
| Lysmd2 | 2.5933 | 0.0003 | up | -1.8494 | 0.0000 | down |
| Anxa2 | 1.9256 | 0.0118 | up | -1.3008 | 0.0348 | down |
| Cyp1a2 | -2.2894 | 0.0018 | down | 2.1374 | 0.0018 | up |
| Cdc25a | 1.1908 | 0.0294 | up | -1.4051 | 0.0000 | down |
| Ablim3 | -1.3016 | 0.0096 | down | 1.6567 | 0.0000 | up |
| Cyp2c38 | -5.1022 | 0.0000 | down | 3.0120 | 0.0000 | up |
| Rims3 | -2.7357 | 0.0001 | down | 1.2605 | 0.0221 | up |
| Pygb | 1.8151 | 0.0242 | up | -1.5650 | 0.0006 | down |
| Nat8f2 | -1.7068 | 0.0000 | down | 1.3564 | 0.0001 | up |
| Ypel4 | 3.0763 | 0.0040 | up | -3.0612 | 0.0014 | down |
| Myo15b | 3.2927 | 0.0000 | up | -2.6341 | 0.0002 | down |
| Gulo | -1.0407 | 0.0074 | down | 0.7324 | 0.0462 | up |
| Hsd17b13 | 1.6123 | 0.0005 | up | -0.8493 | 0.0467 | down |
| Ly6d | 3.3475 | 0.0005 | up | -3.0368 | 0.0004 | down |
| Cdhr2 | 2.6192 | 0.0028 | up | -2.0733 | 0.0324 | down |
| Stab2 | -2.2931 | 0.0000 | down | 1.9372 | 0.0000 | up |
| Aldh1b1 | 2.0702 | 0.0054 | up | -0.8555 | 0.0170 | down |
| Frmpd1 | -6.8122 | 0.0054 | down | 6.9333 | 0.0001 | up |
| Gramd1c | -1.5277 | 0.0415 | down | 1.2095 | 0.0007 | up |
| Nodal | 1.9334 | 0.0179 | up | -2.5036 | 0.0001 | down |
| Spon2 | 2.3950 | 0.0000 | up | -0.9341 | 0.0090 | down |
| Cib2 | -2.6001 | 0.0043 | down | 1.7495 | 0.0240 | up |
| Pcp4l1 | -1.6962 | 0.0157 | down | 1.2896 | 0.0035 | up |
| Egr1 | 2.0351 | 0.0000 | up | -1.9036 | 0.0000 | down |
| Cyp3a16 | -5.6185 | 0.0361 | down | 3.5181 | 0.0000 | up |
| Ifi203 | -1.7920 | 0.0281 | down | 1.5577 | 0.0003 | up |
| Elapor1 | 3.1410 | 0.0001 | up | -1.3000 | 0.0021 | down |
| Cyp2b13 | -5.9547 | 0.0008 | down | 3.1716 | 0.0000 | up |
| Mthfd1l | 1.9660 | 0.0047 | up | -1.2813 | 0.0023 | down |
| Kcna2 | -3.8799 | 0.0008 | down | 2.6416 | 0.0041 | up |
| Atp1b2 | -4.1258 | 0.0182 | down | 2.5460 | 0.0063 | up |
| Serpina5 | -2.0712 | 0.0185 | down | 1.9666 | 0.0242 | up |
| Slco1a1 | -3.3951 | 0.0000 | down | 2.0342 | 0.0328 | up |
| Cyp2c37 | -3.0495 | 0.0000 | down | 3.0650 | 0.0000 | up |
| Cux2 | -6.4355 | 0.0029 | down | 4.0860 | 0.0000 | up |
| Notum | -1.5870 | 0.0039 | down | 1.9787 | 0.0000 | up |
| Filip1l | -1.8534 | 0.0242 | down | 1.1155 | 0.0008 | up |
| Lrit2 | -2.9614 | 0.0029 | down | 2.9945 | 0.0003 | up |
| Ncmap | -4.8583 | 0.0165 | down | 4.5761 | 0.0002 | up |
| Arhgef37 | -1.8463 | 0.0015 | down | 1.7401 | 0.0004 | up |
| Lrtm1 | -4.1488 | 0.0000 | down | 3.2825 | 0.0000 | up |
| Slc25a51 | -1.1979 | 0.0143 | down | 1.3035 | 0.0002 | up |
| Igfals | -1.9386 | 0.0273 | down | 1.7790 | 0.0000 | up |
| Chrm3 | 4.6190 | 0.0000 | up | -2.2548 | 0.0001 | down |
| Sned1 | -1.7083 | 0.0254 | down | 2.4196 | 0.0000 | up |
| Cd300e | -2.7063 | 0.0281 | down | 1.0872 | 0.0458 | up |
| Sprr1a | 3.2459 | 0.0242 | up | -2.4887 | 0.0447 | down |
| Cyp8b1 | -3.2934 | 0.0004 | down | 3.8983 | 0.0000 | up |
| Acot4 | 1.1158 | 0.0116 | up | 1.6585 | 0.0000 | up |
| Slc22a26 | -4.2823 | 0.0000 | down | 2.3424 | 0.0021 | up |
| Cyp2c50 | -3.7672 | 0.0002 | down | 4.0108 | 0.0038 | up |
| Zfp811 | -1.7750 | 0.0440 | down | 1.4906 | 0.0295 | up |
| Wdr86 | -3.2512 | 0.0354 | down | 1.5917 | 0.0305 | up |
| Timd4 | -3.4861 | 0.0000 | down | 1.6899 | 0.0077 | up |
| Gm10032 | 3.7505 | 0.0344 | up | -1.6741 | 0.0425 | down |
| Tubb2a | 1.4190 | 0.0278 | up | -1.8452 | 0.0000 | down |
| Ces3b | -3.3312 | 0.0000 | down | 3.1916 | 0.0000 | up |
| Cyp26b1 | -3.0225 | 0.0068 | down | 2.9564 | 0.0021 | up |
| mt-Co3 | -1.8377 | 0.0380 | down | 1.2954 | 0.0214 | up |
| Cyp4a10 | 2.4195 | 0.0192 | up | 1.8541 | 0.0000 | up |
| Cyp2c54 | -4.3678 | 0.0000 | down | 4.5543 | 0.0000 | up |
| Lilra5 | -2.4450 | 0.0216 | down | 1.4207 | 0.0011 | up |
| Naip5 | -1.7089 | 0.0415 | down | 1.2436 | 0.0021 | up |
| Sh3d21 | -2.4538 | 0.0011 | down | 2.5949 | 0.0000 | up |
| Sp5 | -2.4819 | 0.0018 | down | 1.6372 | 0.0199 | up |
| Cyp3a41b | -8.7620 | 0.0000 | down | 5.6486 | 0.0000 | up |
| Ces1b | -1.3944 | 0.0175 | down | 1.5193 | 0.0000 | up |
| H2-Q1 | -2.5170 | 0.0315 | down | 1.1371 | 0.0129 | up |
| Gm12909 | -2.3416 | 0.0380 | down | 1.5458 | 0.0251 | up |
| Gm2788 | -2.9037 | 0.0079 | down | 2.6595 | 0.0000 | up |
| Xist | -10.4413 | 0.0000 | down | 11.1402 | 0.0000 | up |
| Gm15675 | -1.9954 | 0.0204 | down | 2.3392 | 0.0000 | up |
| A630031M04Rik | -2.4560 | 0.0237 | down | 3.5839 | 0.0000 | up |
| Tbx3os1 | -3.3119 | 0.0001 | down | 3.0291 | 0.0000 | up |
| Gm15889 | 2.3520 | 0.0001 | up | -1.1769 | 0.0275 | down |
| Gm19705 | 3.0839 | 0.0012 | up | -3.4627 | 0.0000 | down |
| 4732463B04Rik | 1.5438 | 0.0325 | up | 1.0222 | 0.0351 | up |
| Tcf24 | 1.8265 | 0.0133 | up | -1.2183 | 0.0031 | down |
| Gm6135 | -3.0836 | 0.0198 | down | 3.2403 | 0.0006 | up |
| Gm44992 | -3.7217 | 0.0004 | down | 4.5709 | 0.0000 | up |
| Gm4756 | -2.3234 | 0.0001 | down | 1.2799 | 0.0228 | up |
| Gm45470 | -2.3291 | 0.0002 | down | 2.3796 | 0.0000 | up |
| Gm32511 | -2.2809 | 0.0038 | down | 1.6471 | 0.0009 | up |
| Gm32872 | -3.6221 | 0.0093 | down | 2.3822 | 0.0433 | up |
| Gm36264 | -2.8793 | 0.0195 | down | 2.7473 | 0.0000 | up |
| Gm35164 | -3.9227 | 0.0074 | down | 3.1491 | 0.0241 | up |
| Gm49012 | -6.6216 | 0.0036 | down | 8.2834 | 0.0000 | up |
| ENSMUSG00000120337 | -6.1840 | 0.0244 | down | 5.7386 | 0.0100 | up |
| ENSMUSG00000120422 | 1.9065 | 0.0339 | up | -1.7000 | 0.0041 | down |
| ENSMUSG00000121445 | -2.1620 | 0.0000 | down | 2.2588 | 0.0000 | up |

Table S9 The detailed information on shared DEPs

| Protein IDs | Entry Name | Model vs. Control | | | ILW-H vs. Model | | |
| --- | --- | --- | --- | --- | --- | --- | --- |
|  |  | Fold Change | P-value | Trend | Fold Change | P-value | Trend |
| A0A075B5W6 | Ighv1-55 | 0.2950 | 0.0293 | down | 1.5110 | 0.0347 | up |
| A0A087WQN7 | Xrn1 | 0.4358 | 0.0247 | down | 1.7196 | 0.0245 | up |
| A0A087WSB3 | Pira13 | 23.0845 | 0.0000 | up | 0.0433 | 0.0000 | down |
| A0A087WSN6 | Fn1 | 2.1903 | 0.0104 | up | 0.5754 | 0.0405 | down |
| A0A0R4J1B4 | Itgam | 59.5273 | 0.0000 | up | 0.0168 | 0.0000 | down |
| A2A8L1 | Chd5 | 0.0396 | 0.0000 | down | 25.8295 | 0.0000 | up |
| A2A8L5 | Ptprf | 0.5676 | 0.0337 | down | 1.4926 | 0.0037 | up |
| A2AAJ9 | Obscn | 0.0148 | 0.0000 | down | 52.1438 | 0.0000 | up |
| A2AJL3 | Fggy | 0.6962 | 0.0094 | down | 1.4060 | 0.0469 | up |
| D3Z7F6 | Cyp2t4 | 0.2323 | 0.0124 | down | 2.3286 | 0.0475 | up |
| D9J2Z9 | Pdlim5 | 9.1576 | 0.0000 | up | 0.1092 | 0.0000 | down |
| E0CYR6 | Nat8f7 | 0.0301 | 0.0000 | down | 38.7304 | 0.0000 | up |
| E9PVB8 | Fgd2 | 21.4480 | 0.0000 | up | 0.0466 | 0.0000 | down |
| E9PX96 | Sva | 243.4304 | 0.0000 | up | 0.0041 | 0.0000 | down |
| E9Q394 | Akap13 | 1.4768 | 0.0482 | up | 0.6971 | 0.0415 | down |
| F2Z472 | Svs3a | 248.8149 | 0.0000 | up | 0.0040 | 0.0000 | down |
| F6T8X6 | Sec16a | 0.6100 | 0.0426 | down | 1.8511 | 0.0453 | up |
| O08524 | Tectb | 0.2755 | 0.0000 | down | 3.5409 | 0.0000 | up |
| O08966 | Slc22a1 | 0.4277 | 0.0138 | down | 1.7285 | 0.0458 | up |
| O08992 | Sdcbp | 2.0948 | 0.0089 | up | 0.5931 | 0.0118 | down |
| O09030 | Il13ra1 | 0.2923 | 0.0000 | down | 3.9778 | 0.0000 | up |
| O09161 | Casq2 | 3.8521 | 0.0000 | up | 0.2596 | 0.0000 | down |
| O09165 | Casq1 | 0.1618 | 0.0000 | down | 12.1949 | 0.0000 | up |
| O35127 | Grcc10 | 1.4637 | 0.0084 | up | 0.7249 | 0.0038 | down |
| O35536 | Tfpi2 | 0.0119 | 0.0000 | down | 64.5978 | 0.0000 | up |
| O35648 | Cetn3 | 3.6702 | 0.0000 | up | 0.2725 | 0.0000 | down |
| O35737 | Hnrnph1 | 1.6967 | 0.0031 | up | 0.8040 | 0.0490 | down |
| O70468 | Mybpc3 | 0.1434 | 0.0000 | down | 2.3307 | 0.0000 | up |
| O70551 | Srpk1 | 1.3813 | 0.0201 | up | 0.7599 | 0.0338 | down |
| O88667 | Rrad | 21.5064 | 0.0000 | up | 0.0465 | 0.0000 | down |
| O88833 | Cyp4a10 | 3.2685 | 0.0163 | up | 2.1463 | 0.0310 | up |
| O88962 | Cyp8b1 | 0.3245 | 0.0484 | down | 2.9977 | 0.0294 | up |
| O89029 | Matn4 | 212.1939 | 0.0000 | up | 0.0047 | 0.0000 | down |
| P00186 | Cyp1a2 | 0.3437 | 0.0260 | down | 2.0175 | 0.0183 | up |
| P01897 | H2-L | 0.0015 | 0.0000 | down | 170.7348 | 0.0000 | up |
| P01910 | H2-Aa | 19.4600 | 0.0000 | up | 0.0514 | 0.0000 | down |
| P06342 | H2-Ab1 | 0.0504 | 0.0000 | down | 14.0146 | 0.0000 | up |
| P09470 | Ace | 28.6413 | 0.0000 | up | 0.4034 | 0.0151 | down |
| P09671 | Sod2 | 0.7951 | 0.0349 | down | 1.2773 | 0.0392 | up |
| P09803 | Cdh1 | 2.3572 | 0.0002 | up | 0.6535 | 0.0024 | down |
| P09925 | Surf1 | 0.7932 | 0.0429 | down | 1.2409 | 0.0486 | up |
| P0DJE0 | Pet100 | 0.5692 | 0.0285 | down | 2.8929 | 0.0220 | up |
| P11798 | Camk2a | 5.6795 | 0.0000 | up | 0.1761 | 0.0000 | down |
| P14069 | S100a6 | 2.2193 | 0.0306 | up | 0.5061 | 0.0232 | down |
| P16882 | Ghr | 0.0519 | 0.0000 | down | 21.6481 | 0.0000 | up |
| P24721 | Asgr2 | 0.5325 | 0.0052 | down | 1.2385 | 0.0479 | up |
| P26043 | Rdx | 0.7981 | 0.0252 | down | 1.2335 | 0.0320 | up |
| P27546 | Map4 | 1.3590 | 0.0035 | up | 0.8154 | 0.0217 | down |
| P28654 | Dcn | 2.3842 | 0.0241 | up | 0.6269 | 0.0131 | down |
| P28658 | Atxn10 | 1.5413 | 0.0048 | up | 0.7729 | 0.0233 | down |
| P28704 | Rxrb | 0.0192 | 0.0000 | down | 7.8122 | 0.0000 | up |
| P28843 | Dpp4 | 0.5368 | 0.0289 | down | 1.5186 | 0.0127 | up |
| P29387 | Gnb4 | 1.4378 | 0.0012 | up | 0.7562 | 0.0053 | down |
| P31809 | Ceacam1 | 0.6165 | 0.0071 | down | 1.4452 | 0.0340 | up |
| P35456 | Plaur | 43.0647 | 0.0000 | up | 0.0232 | 0.0000 | down |
| P35486 | Pdha1 | 0.7034 | 0.0179 | down | 1.2932 | 0.0235 | up |
| P46096 | Syt1 | 0.0618 | 0.0000 | down | 22.3716 | 0.0000 | up |
| P47199 | Cryz | 0.7113 | 0.0470 | down | 1.3813 | 0.0298 | up |
| P48725 | Pcnt | 11.0391 | 0.0000 | up | 0.0906 | 0.0000 | down |
| P50171 | Hsd17b8 | 0.6714 | 0.0063 | down | 1.4082 | 0.0276 | up |
| P51125 | Cast | 1.3948 | 0.0164 | up | 0.8196 | 0.0083 | down |
| P56212 | Arpp19 | 1.4375 | 0.0126 | up | 0.7711 | 0.0429 | down |
| P56395 | Cyb5a | 0.6797 | 0.0041 | down | 1.2694 | 0.0239 | up |
| P56656 | Cyp2c39 | 0.2703 | 0.0006 | down | 3.2782 | 0.0010 | up |
| P62492 | Rab11a | 9.7487 | 0.0000 | up | 0.1026 | 0.0000 | down |
| P62700 | Ypel5 | 1.4403 | 0.0250 | up | 0.5565 | 0.0053 | down |
| P62984 | Uba52 | 0.0904 | 0.0000 | down | 15.6015 | 0.0000 | up |
| P70388 | Rad50 | 1.7972 | 0.0192 | up | 0.7584 | 0.0411 | down |
| P84075 | Hpca | 8.4154 | 0.0000 | up | 0.1188 | 0.0000 | down |
| P97411 | Ica1 | 14.3062 | 0.0000 | up | 0.0699 | 0.0000 | down |
| Q00420 | Gabpb1 | 0.4504 | 0.0423 | down | 0.9539 | 0.0459 | down |
| Q00547 | Hmmr | 34.1192 | 0.0000 | up | 0.0293 | 0.0000 | down |
| Q05CK8 | Ube2d3 | 0.0993 | 0.0000 | down | 32.0825 | 0.0000 | up |
| Q07916 | Pou6f1 | 0.1008 | 0.0000 | down | 4.2141 | 0.0000 | up |
| Q08639 | Tfdp1 | 4.0000 | 0.0000 | up | 0.2500 | 0.0000 | down |
| Q09098 | Pate4 | 85.4442 | 0.0000 | up | 0.0117 | 0.0000 | down |
| Q0VGT4 | Zgrf1 | 0.0091 | 0.0000 | down | 65.2076 | 0.0000 | up |
| Q3TUF7 | Yeats2 | 4.7158 | 0.0000 | up | 0.2121 | 0.0000 | down |
| Q3UGR5 | Hdhd2 | 0.8062 | 0.0328 | down | 1.3225 | 0.0220 | up |
| Q3UN54 | Pate14 | 14.4030 | 0.0000 | up | 0.0694 | 0.0000 | down |
| Q3UQU0 | Brd9 | 0.0776 | 0.0000 | down | 21.4243 | 0.0000 | up |
| Q3UU96 | Cdc42bpa | 0.0372 | 0.0000 | down | 25.5270 | 0.0000 | up |
| Q4QRL3 | Ccdc88b | 14.3181 | 0.0000 | up | 0.0698 | 0.0000 | down |
| Q4VAE3 | Tmem65 | 0.1348 | 0.0000 | down | 13.6535 | 0.0000 | up |
| Q5EBG8 | -- | 0.7522 | 0.0211 | down | 1.3370 | 0.0324 | up |
| Q5SUV2 | Mycbpap | 0.0733 | 0.0000 | down | 26.3956 | 0.0000 | up |
| Q60973 | Rbbp7 | 1.5244 | 0.0079 | up | 0.7572 | 0.0207 | down |
| Q61029 | Tmpo | 1.3518 | 0.0123 | up | 0.8253 | 0.0383 | down |
| Q61127 | Nab2 | 10.0745 | 0.0000 | up | 0.0993 | 0.0000 | down |
| Q61526 | Erbb3 | 0.4501 | 0.0098 | down | 2.5517 | 0.0016 | up |
| Q62376 | Snrnp70 | 1.2955 | 0.0255 | up | 0.7807 | 0.0287 | down |
| Q63886 | Ugt1a1 | 0.5903 | 0.0043 | down | 2.0017 | 0.0010 | up |
| Q64356 | Svs6 | 125.0251 | 0.0000 | up | 0.0080 | 0.0000 | down |
| Q64442 | Sord | 0.6485 | 0.0435 | down | 1.5820 | 0.0306 | up |
| Q64505 | Cyp7a1 | 0.4105 | 0.0398 | down | 4.9876 | 0.0102 | up |
| Q64732 | Foxb1 | 0.1767 | 0.0000 | down | 4.8097 | 0.0000 | up |
| Q68FM7 | Arhgef11 | 8.4048 | 0.0000 | up | 0.1190 | 0.0000 | down |
| Q69ZR2 | Hectd1 | 1.2800 | 0.0468 | up | 0.7389 | 0.0146 | down |
| Q6NXK2 | Znf532 | 3.4164 | 0.0000 | up | 0.2927 | 0.0000 | down |
| Q6P1F6 | Ppp2r2a | 1.4057 | 0.0132 | up | 0.8209 | 0.0145 | down |
| Q6P1Y1 | Rtl3 | 91.3673 | 0.0000 | up | 0.0109 | 0.0000 | down |
| Q6P4U0 | Thsd7b | 0.1861 | 0.0000 | down | 6.8111 | 0.0000 | up |
| Q6PAM1 | Txlna | 1.2082 | 0.0386 | up | 0.8318 | 0.0383 | down |
| Q6PDQ2 | Chd4 | 1.2215 | 0.0028 | up | 0.7630 | 0.0043 | down |
| Q6ZQM8 | Ugt1a7 | 3.2041 | 0.0021 | up | 0.1874 | 0.0292 | down |
| Q6ZWY8 | Tmsb10 | 1.6103 | 0.0274 | up | 0.6735 | 0.0434 | down |
| Q78ZA7 | Nap1l4 | 1.2351 | 0.0461 | up | 0.7569 | 0.0166 | down |
| Q7TMB8 | Cyfip1 | 1.3131 | 0.0331 | up | 0.7534 | 0.0312 | down |
| Q80U44 | Zfyve16 | 0.1139 | 0.0000 | down | 8.5324 | 0.0000 | up |
| Q80UG2 | Plxna4 | 8.1543 | 0.0000 | up | 0.1226 | 0.0000 | down |
| Q80VL1 | Tdrkh | 0.1806 | 0.0000 | down | 3.3937 | 0.0000 | up |
| Q80YV3 | Trrap | 11.0719 | 0.0000 | up | 0.0903 | 0.0000 | down |
| Q8BGA5 | Krr1 | 2.1302 | 0.0234 | up | 0.5588 | 0.0281 | down |
| Q8BGX0 | Trim23 | 0.0037 | 0.0000 | down | 511.9734 | 0.0000 | up |
| Q8BH00 | Aldh8a1 | 0.7039 | 0.0488 | down | 1.4266 | 0.0401 | up |
| Q8BH61 | F13a1 | 2.1283 | 0.0100 | up | 0.5242 | 0.0184 | down |
| Q8BHG1 | Nrdc | 1.4362 | 0.0194 | up | 0.8030 | 0.0139 | down |
| Q8BHX1 | Haus1 | 0.0056 | 0.0000 | down | 290.8018 | 0.0000 | up |
| Q8BKI2 | Tnrc6b | 1.2894 | 0.0464 | up | 0.7120 | 0.0194 | down |
| Q8BM26 | Lyz3 | 12.4646 | 0.0000 | up | 0.0802 | 0.0000 | down |
| Q8BMF4 | Dlat | 0.7126 | 0.0045 | down | 1.2496 | 0.0126 | up |
| Q8BMS1 | Hadha | 0.8284 | 0.0327 | down | 1.4219 | 0.0161 | up |
| Q8BP40 | Acp6 | 0.7163 | 0.0110 | down | 1.2945 | 0.0309 | up |
| Q8BTS4 | Nup54 | 1.2570 | 0.0211 | up | 0.7560 | 0.0312 | down |
| Q8BUR9 | Mzt1 | 0.0666 | 0.0000 | down | 15.7060 | 0.0000 | up |
| Q8BYI8 | Fam234b | 0.0064 | 0.0000 | down | 196.1555 | 0.0000 | up |
| Q8BZ09 | Slc25a21 | 0.5134 | 0.0048 | down | 1.6076 | 0.0005 | up |
| Q8BZH8 | Svs3b | 375.0554 | 0.0000 | up | 0.0027 | 0.0000 | down |
| Q8C0K5 | Slc25a16 | 0.6737 | 0.0101 | down | 1.2480 | 0.0396 | up |
| Q8C147 | Dock8 | 2.0793 | 0.0266 | up | 0.2410 | 0.0456 | down |
| Q8CDS7 | Fam243 | 0.1348 | 0.0000 | down | 8.0670 | 0.0000 | up |
| Q8CFE3 | Rcor1 | 1.4168 | 0.0241 | up | 0.6952 | 0.0309 | down |
| Q8CI51 | Pdlim5 | 1.3091 | 0.0124 | up | 0.8332 | 0.0405 | down |
| Q8CIH9 | Ppat | 1.6489 | 0.0239 | up | 0.7853 | 0.0068 | down |
| Q8K0V2 | Dcun1d3 | 7.5140 | 0.0000 | up | 0.1331 | 0.0000 | down |
| Q8K400 | Stxbp5 | 1.6756 | 0.0168 | up | 0.5318 | 0.0142 | down |
| Q8K4E0 | Alms1 | 5.9577 | 0.0000 | up | 0.1679 | 0.0000 | down |
| Q8R086 | Suox | 0.5691 | 0.0439 | down | 1.8656 | 0.0322 | up |
| Q8R0S1 | Atf7 | 0.5403 | 0.0397 | down | 0.5259 | 0.0409 | down |
| Q8R164 | Bphl | 0.6185 | 0.0129 | down | 1.5296 | 0.0246 | up |
| Q8R1F9 | Rpp40 | 0.1931 | 0.0000 | down | 16.8199 | 0.0000 | up |
| Q8R1T4 | Slc35a3 | 0.6986 | 0.0431 | down | 0.7286 | 0.0498 | down |
| Q8R3Q2 | Ppp6r2 | 0.0440 | 0.0000 | down | 17.3482 | 0.0000 | up |
| Q8R5J9 | Arl6ip5 | 2.1900 | 0.0032 | up | 0.4779 | 0.0051 | down |
| Q8VC19 | Alas1 | 0.6127 | 0.0104 | down | 1.6737 | 0.0128 | up |
| Q8VCA8 | Scrn2 | 0.6572 | 0.0172 | down | 1.4380 | 0.0207 | up |
| Q8VCM3 | Zfyve21 | 16.9747 | 0.0000 | up | 0.0589 | 0.0000 | down |
| Q8VCR7 | Abhd14b | 0.6247 | 0.0182 | down | 1.3430 | 0.0328 | up |
| Q8VHH5 | Agap3 | 9.0658 | 0.0000 | up | 0.1103 | 0.0000 | down |
| Q8WUR0 | -- | 0.6778 | 0.0268 | down | 1.2632 | 0.0199 | up |
| Q91VA0 | Acsm1 | 0.4116 | 0.0012 | down | 1.6044 | 0.0027 | up |
| Q91VM9 | Ppa2 | 0.7417 | 0.0146 | down | 1.3670 | 0.0055 | up |
| Q91VT4 | Cbr4 | 0.6571 | 0.0022 | down | 1.3529 | 0.0063 | up |
| Q91WA1 | Tipin | 3.2510 | 0.0000 | up | 0.3076 | 0.0000 | down |
| Q91WN1 | Dnajc9 | 1.9887 | 0.0053 | up | 0.6717 | 0.0390 | down |
| Q91XA2 | Golm1 | 3.6707 | 0.0168 | up | 0.6150 | 0.0109 | down |
| Q921J4 | Ube2s | 2.8758 | 0.0000 | up | 0.3477 | 0.0000 | down |
| Q922B1 | Macrod1 | 0.4495 | 0.0120 | down | 2.1462 | 0.0157 | up |
| Q923B0 | Ggact | 0.6797 | 0.0439 | down | 1.5978 | 0.0294 | up |
| Q924Y0 | Bbox1 | 0.8048 | 0.0252 | down | 1.3652 | 0.0052 | up |
| Q925H1 | Trps1 | 4.4245 | 0.0000 | up | 0.2260 | 0.0000 | down |
| Q99J39 | Mlycd | 0.6907 | 0.0000 | down | 1.2752 | 0.0298 | up |
| Q99K43 | Prc1 | 13.7386 | 0.0000 | up | 0.0728 | 0.0000 | down |
| Q99L04 | Dhrs1 | 0.6604 | 0.0133 | down | 1.3559 | 0.0333 | up |
| Q99M31 | Hspa14 | 1.3134 | 0.0149 | up | 0.7654 | 0.0177 | down |
| Q99MD6 | Txnrd3 | 0.0558 | 0.0000 | down | 11.7298 | 0.0000 | up |
| Q99N50 | Sytl2 | 0.0008 | 0.0000 | down | 1514.5730 | 0.0000 | up |
| Q99NE5 | Rims1 | 0.1207 | 0.0000 | down | 27.5363 | 0.0000 | up |
| Q99NF1 | Bco2 | 0.6330 | 0.0080 | down | 1.4455 | 0.0321 | up |
| Q99PI4 | Lpin3 | 3.0603 | 0.0000 | up | 0.3268 | 0.0000 | down |
| Q9CPR4 | Rpl17 | 0.8161 | 0.0328 | down | 0.8096 | 0.0275 | down |
| Q9CPW2 | Fdx2 | 0.6911 | 0.0475 | down | 1.4918 | 0.0445 | up |
| Q9CQW1 | Ykt6 | 1.4214 | 0.0246 | up | 0.7469 | 0.0432 | down |
| Q9CX80 | Cygb | 2.7464 | 0.0030 | up | 0.6191 | 0.0412 | down |
| Q9CZ42 | Naxd | 0.7511 | 0.0230 | down | 1.3175 | 0.0055 | up |
| Q9D114 | Hddc3 | 0.7802 | 0.0389 | down | 1.3269 | 0.0273 | up |
| Q9D142 | Nudt14 | 0.6620 | 0.0273 | down | 1.5401 | 0.0208 | up |
| Q9D172 | Gatd3 | 0.7303 | 0.0337 | down | 1.4420 | 0.0212 | up |
| Q9D4V7 | Rabl3 | 0.0027 | 0.0000 | down | 619.9393 | 0.0000 | up |
| Q9D5J6 | Shpk | 0.7022 | 0.0167 | down | 1.2504 | 0.0094 | up |
| Q9D6E4 | Ttc9b | 0.0271 | 0.0000 | down | 21.2687 | 0.0000 | up |
| Q9D6K5 | Synj2bp | 0.5891 | 0.0092 | down | 1.5109 | 0.0323 | up |
| Q9D7B6 | Acad8 | 0.7563 | 0.0346 | down | 1.2892 | 0.0448 | up |
| Q9D7S9 | Chmp5 | 1.4171 | 0.0024 | up | 0.6551 | 0.0014 | down |
| Q9D8S3 | Arfgap3 | 1.2130 | 0.0426 | up | 0.7976 | 0.0264 | down |
| Q9D975 | Srxn1 | 2.3992 | 0.0392 | up | 0.4525 | 0.0316 | down |
| Q9DBG9 | Tax1bp3 | 1.8499 | 0.0413 | up | 0.6910 | 0.0295 | down |
| Q9DCG6 | Pbld1 | 0.5110 | 0.0057 | down | 1.8861 | 0.0378 | up |
| Q9EP96 | Slco1a4 | 0.7074 | 0.0421 | down | 2.3130 | 0.0224 | up |
| Q9EPW0 | Inpp4a | 0.0787 | 0.0000 | down | 4.8313 | 0.0000 | up |
| Q9EQ20 | Aldh6a1 | 0.6841 | 0.0305 | down | 1.4234 | 0.0080 | up |
| Q9EQ28 | Pold3 | 8.5486 | 0.0000 | up | 0.1170 | 0.0000 | down |
| Q9EQP2 | Ehd4 | 1.5052 | 0.0341 | up | 0.6627 | 0.0349 | down |
| Q9EQQ9 | Oga | 1.6885 | 0.0466 | up | 0.7516 | 0.0394 | down |
| Q9ERG2 | Strn3 | 1.2882 | 0.0230 | up | 0.7899 | 0.0296 | down |
| Q9ES81 | Popdc3 | 142.1474 | 0.0000 | up | 0.0070 | 0.0000 | down |
| Q9JHS9 | Cwc15 | 1.2506 | 0.0414 | up | 1.4212 | 0.0209 | up |
| Q9JIL4 | Pdzk1 | 0.7447 | 0.0180 | down | 1.4397 | 0.0090 | up |
| Q9JIY5 | Htra2 | 0.8014 | 0.0250 | down | 1.4481 | 0.0055 | up |
| Q9JKK8 | Atr | 5.6420 | 0.0000 | up | 0.1772 | 0.0000 | down |
| Q9JL16 | Isg20 | 8.9635 | 0.0000 | up | 0.1116 | 0.0000 | down |
| Q9JL62 | Gltp | 2.1979 | 0.0065 | up | 0.7035 | 0.0220 | down |
| Q9QUG9 | Rasgrp2 | 0.7596 | 0.0245 | down | 0.0768 | 0.0000 | down |
| Q9QUJ7 | Acsl4 | 1.8001 | 0.0343 | up | 0.5526 | 0.0009 | down |
| Q9QUK6 | Tlr4 | 31.0663 | 0.0000 | up | 0.0322 | 0.0000 | down |
| Q9QXY6 | Ehd3 | 0.7949 | 0.0025 | down | 1.2618 | 0.0024 | up |
| Q9QYR6 | Map1a | 19.1179 | 0.0000 | up | 0.0523 | 0.0000 | down |
| Q9R0Q6 | Arpc1a | 0.6319 | 0.0028 | down | 1.2859 | 0.0256 | up |
| Q9R1K5 | Fzr1 | 10.9373 | 0.0000 | up | 0.0914 | 0.0000 | down |
| Q9R1Q9 | Atp6ap1 | 1.5436 | 0.0293 | up | 0.6128 | 0.0099 | down |
| Q9R1S7 | Abcc6 | 0.6925 | 0.0018 | down | 1.2332 | 0.0435 | up |
| Q9WTP7 | Ak3 | 0.7287 | 0.0057 | down | 1.4133 | 0.0033 | up |
| Q9WUD0 | Cyp2b10 | 1.4930 | 0.0365 | up | 2.2066 | 0.0037 | up |
| Q9WUR2 | Eci2 | 0.6782 | 0.0015 | down | 1.3130 | 0.0114 | up |
| Q9WVL0 | Gstz1 | 0.6623 | 0.0192 | down | 1.3808 | 0.0341 | up |
| Q9Z0U0 | Xpr1 | 0.1843 | 0.0000 | down | 19.6378 | 0.0000 | up |
| Q9Z1K8 | Slc7a7 | 6.5453 | 0.0000 | up | 0.1528 | 0.0000 | down |
| Q9Z2L6 | Minpp1 | 1.2980 | 0.0237 | up | 0.6124 | 0.0482 | down |
| Q9Z2Y8 | Plpbp | 0.7797 | 0.0422 | down | 1.2608 | 0.0096 | up |
| Q9Z351 | Kcnq2 | 0.0878 | 0.0000 | down | 9.5630 | 0.0000 | up |
| S4R1I9 | Zfp715 | 5.4882 | 0.0000 | up | 0.1822 | 0.0000 | down |
